# The residues 4 to 6 at the N-terminus in particular modulate fibril propagation of β _2_-microglobulin: N-terminal modulates β2M fibril propagation

**DOI:** 10.3724/abbs.2021017

**Published:** 2021-12-23

**Authors:** Haibin Dang, Zhixian Chen, Wang Chen, Xudong Luo, Pan Liu, Liqiang Wang, Jie Chen, Xuhai Tang, Zhengzhi Wang, Yi Liang

**Affiliations:** 1 Hubei Key Laboratory of Cell Homeostasis College of Life Sciences Wuhan University Wuhan 430072 China 2.Wuhan University Shenzhen Research Institute Shenzhen 518057 China and 3.School of Civil Engineering Wuhan University Wuhan 430072 China

**Keywords:** human β _2_-microglobulin, amyloid fibril, conformational stability, truncated variant, fibril propagation, atomic force microscopy

## Abstract

The ΔN6 truncation is the main posttranslational modification of β _2_-microglobulin (β _2_M) found in dialysis-related amyloid. Investigation of the interaction of wild-type (WT) β _2_M with N-terminally truncated variants is therefore of medical relevance. However, it is unclear which residues among the six residues at the N-terminus are crucial to the interactions and the modulation of amyloid fibril propagation of β _2_M. We herein analyzed homo- and heterotypic seeding of amyloid fibrils of WT human β _2_M and its N-terminally-truncated variants ΔN1 to ΔN6, lacking up to six residues at the N-terminus. At acidic pH 2.5, we produced amyloid fibrils *in vitro* from recombinant, WT β _2_M and its six truncated variants, and found that ΔN6 β _2_M fibrils exhibit a significantly lower conformational stability than WT β _2_M fibrils. Importantly, under more physiological conditions (pH 6.2), we assembled amyloid fibrils *in vitro* only from recombinant, ΔN4, ΔN5, and ΔN6 β _2_M but not from WT β _2_M and its three truncated variants ΔN1 to ΔN3. Notably, the removal of the six, five or four residues at the N-terminus leads to enhanced fibril formation, and homo- and heterotypic seeding of ΔN6 fibrils strongly promotes amyloid fibril formation of WT β _2_M and its six truncated variants, including at more physiological pH 6.2. Collectively, these results demonstrated that the residues 4 to 6 at the N-terminus particularly modulate amyloid fibril propagation of β _2_M and the interactions of WT β _2_M with N-terminally truncated variants, potentially indicating the direct relevance to the involvement of the protein’s aggregation in dialysis-related amyloidosis.

## Introduction

The misfolding of proteins into insoluble amyloid fibrils causes more than 30 human diseases, such as Alzheimer disease caused by the misfolding of Tau and amyloid β, Parkinson’s disease caused by the misfolding of α-synuclein, and dialysis-related amyloidosis caused by the misfolding of β _2_-microglobulin (β _2_M) [ 1– 6].

Human β _2_M with 99 amino acid residues is the light chain of major histocompatibility complex I (MHC I) [7]. The native state of β _2_M adopts an immunoglobulin fold with seven β-strands (A through G) to form a β-sandwich structure composed of two facing β-sheets, which is stabilized by a disulfide bond linkage at Cys25 in strand B and Cys80 in strand F between the two β-sheets [ 7, 8]. After dissociation from MHC I, it circulates in plasma in a free monomer and is degraded in the kidney [9]. However, the clearance of plasma β _2_M in the kidney disease patients with chronic dialysis treatment is disrupted or disturbed, leading to an increase in the concentration of β _2_M up to 60 folds, reaching ~50–70 μg/mL compared with the normal concentration of 1–2 μg/mL [ 10, 11]. Owing to such high plasma concentrations, amyloid fibrils of β _2_M deposit in the joints and connective tissues, causing dialysis-related amyloidosis [ 10, 12, 13]. Since identification of β _2_M as a dominating component of dialysis-associated amyloidosis [14], a lot of studies have been carried out in the past two decades to clarify the pathogenesis of the disease and aggregation mechanism of β _2_M [ 13, 15– 21]. It has been shown that β _2_M aggregation is accelerated by extracellular matrix components that are rich in the joints *in vitro*, such as collagens, heparin, and polyphosphate [ 13, 22– 24], and by macromolecular crowders [ 25, 26]. Collagen I inhibits the fibril growth of wild-type (WT) β _2_M when incubated with the WT fibril seeds but does not suppress the fibril assembly of WT β _2_M when seeded with ΔN6 seeds [13]. The structure of WT β _2_M fibril at pH 2.5 contains six β-strands and unique π-stacking interactions perpendicular to the fibril axis [27].

Biochemical analyses of amyloid deposition in dialysis-related amyloidosis patients showed that ΔN6, a truncated variant of β _2_M lacking the N-terminal hexapeptide ^1^IQRTPK ^6^, accounts for approximately 30% of the deposition [ 28– 30]. Investigation of the interaction of WT β _2_M with N-terminally truncated variants is therefore of medical relevance. Compared with WT β _2_M [ 13, 26, 31– 34], ΔN6 β _2_M forms fibrils easily at more physiological pH *in vitro* without any cosolvents or additives [ 25, 29, 30, 35] and has a high tendency to form fibrils [ 15, 29, 33, 35– 38]. However, it is unclear which residues among the six residues at the N-terminus are crucial to the interactions and the modulation of amyloid fibril propagation of β _2_M.

In order to bridge the gap between the first six residues and the first residue at the N-terminus, we analyzed homo- and heterotypic seeding of amyloid fibrils of WT human β _2_M and its N-terminally-truncated variants ΔN1 to ΔN6, lacking up to six residues at the N-terminus. Our results indicated that under more physiological conditions (pH 6.2 instead of pH 2.5), the loss of the first six, five or four residues facilitated amyloid fibril formation of β _2_M. What is more, we demonstrated that at more physiological pH (pH 6.2), homo- and heterotypic seeding of ΔN6 fibrils strongly promoted amyloid fibril formation of WT β _2_M and its six truncated variants. Our results provide direct evidence that the residues 4 to 6 at the N-terminus play vital roles in the fibrillization of β _2_M via modulating amyloid fibril propagation of β _2_M and the interactions of WT β _2_M with N-terminally truncated variants.

## Materials and Methods

### Materials

Congo red, thioflavin T (ThT), and guanidine thiocyanate (GdnSCN) were purchased from Sigma-Aldrich (St Louis, USA). DNA polymerase KOD-plus-Neo was from Toyobo (Tokyo, Japan). Sarkosyl and guanidine hydrochloride (GdnHCl) were obtained from Amresco (Solon, USA). Q-Sepharose Fast Flow was purchased from GE Company (Pittsburgh, USA). All other reagents used were made in China and of analytical grade. All reagent solutions used were prepared in either 10 mM NaH _2_PO _4_-H _3_PO _4_ (pH 2.5) or 50 mM MES buffer (pH 6.2) containing 120 mM NaCl unless specified otherwise.

### Plasmid construction and protein purification

A plasmid-encoding wild-type human β _2_M was a kind gift from Dr David Eisenberg (University of California, Los Angeles, USA). The gene for β _2_M was constructed in the vector pET22b, and β _2_M mutants ΔN1, ΔN2, ΔN3, ΔN4, ΔN5, and ΔN6 were constructed by site-directed mutagenesis using a WT β _2_M template. The primers are shown in Supplementary Table S1. All β _2_M plasmids were transformed into *Escherichia coli*. Recombinant WT β _2_M and its mutants were expressed in *E*. *coli* BL21 (DE3) cells (Merck, Darmstadt, Germany) and purified as described previously [ 25, 39]. SDS-PAGE was used to confirm that all the purified human β _2_M proteins were single species. A NanoDrop OneC Microvolume UV-Vis Spectrophotometer (Thermo Fisher Scientific, Waltham, USA) was used to determine the concentrations of WT β _2_M and its six truncated mutants using their absorbance at 280 nm and molar extinction coefficients calculated from the composition of the protein ( http://web.expasy.org/protparam/). When using UV absorption to detect protein concentration, it is mainly related to aromatic residues. Because the residues 1 to 6 at the N-terminus do not have aromatic residues, the deletion of any of these six residues does not have any effect on the molar extinction coefficient of the products.

### Amyloid fibril formation of β _2_M

The freshly purified β _2_M proteins, which are very stable in pure water, were dialyzed into Mini-Q water and centrifuged at 17,000 *g* and 4°C for 30 min as described previously [ 16, 25]. Then, to form fibrils, WT β _2_M and its six truncated mutants were diluted into 10 mM NaH _2_PO _4_-H _3_PO _4_ (pH 2.5) containing 0.03% NaN _3_ or 50 mM MES buffer (pH 6.2) containing 120 mM NaCl and 0.03% NaN _3_ with a final concentration of 85 μM and incubated at 37°C with a constant stirring rate at 220 rpm. The same concentrations of WT β _2_M and its truncated mutants were maintained by controlling the dilution volume. NaN _3_, used as an antibacterial and antimicrobial agent in stock solutions, did not have any effect on β _2_M proteins. Because β _2_M proteins are very stable, we did not add glycerol to prevent protein aggregation during protein preservation. After different time intervals, aliquots of samples were taken out for thioflavin T (ThT) binding, Congo red binding, atomic force microscopy (AFM), and transmission electron microscopy (TEM) measurements.

### Preparation of β _2_M fibril seeds

Matured fibrils formed from WT β _2_M and its truncated variant ΔN6 β _2_M in the presence of 10 mM NaH _2_PO _4_-H _3_PO _4_ (pH 2.5) or those formed from ΔN6 β _2_M in the presence of 50 mM MES buffer (pH 6.2) containing 120 mM NaCl, all with agitation at 220 rpm and 37 °C, were subjected to ultrasonic fragmentation with a constant power of 300 W and time of 3 s for 3 times. The buffer or pH of matured fibrils formed from WT β _2_M and ΔN6 β _2_M is the same except that WT β _2_M is unable to form matured fibrils in buffers at pH 6.2 and it does not have any effect on the experiments.

### Seed-dependent propagation of β _2_M

The prepared β _2_M was centrifuged at 17,000 *g* and 4 °C for 30 min. The 2% (v/v) of WT β _2_M fibril seeds (or 2% ΔN6 fibril seeds) formed at pH 2.5 were added into solutions of WT β _2_M and its six truncated mutants in 10 mM NaH _2_PO _4_-H _3_PO _4_ (pH 2.5) containing 0.03% NaN _3_ with a final concentration of 85 μM. The 10% (v/v) of ΔN6 fibril seeds produced at pH 6.2 were added into solutions of WT β _2_M and its six truncated mutants in 50 mM MES buffer (pH 6.2) containing 120 mM NaCl and 0.03% NaN _3_ with a final concentration of 85 μM. Then the solutions were incubated at 37°C with a constant stirring rate at 220 rpm. After different time intervals, aliquots of samples were taken out for ThT binding, circular dichroism (CD), ultracentrifugation, TEM, and Cong red binding experiments.

### ThT binding assays

ThT stock solution was freshly prepared in 20 mM NaH _2_PO _4_-Na _2_HPO _4_ buffer (pH 7.4) with the final concentration of 3 mM and passed through a 0.22 μm pore size filter membrane before use to remove insoluble particles. For kinetics of amyloid formation of β _2_M and the seed-dependent propagation of β _2_M, 10 μL of β _2_M fibrils and 5 μL of ThT solution were diluted into 20 mM NaH _2_PO _4_-Na _2_HPO _4_ buffer (pH 7.4), giving a final total volume of 0.6 mL and final concentrations of 1.42 μM for fibrils and 25 μM for ThT. The ThT fluorescence experiments were performed on an LS-55 luminescence spectrometer (PerkinElmer Life Sciences, Shelton, USA) with an excitation wavelength at 450 nm, an emission wavelength at 480 nm (both slit-widths were 5 nm), and the fluorescence voltage at 700 V. Kinetic experiments were repeated at least 3 times at 37°C and measurements were made at 25°C. Our control experiments demonstrated that 25 μM ThT did not quench the fluorescence of 1.42 μM β _2_M fibrils ( Supplementary Figure S1). Kinetic parameters were determined by fitting ThT fluorescence intensity versus time to a sigmoidal equation [ 25, 40– 42]. The time-dependent appearance of seed-dependent propagation was found to be well described by the empirical Hill function equation [43].

### Global denaturation of ΔN6 to ΔN1 fibrils and WT β _2_M fibrils analyzed by ThT fluorescence

Amyloid fibrils (12 μL) were produced from WT β _2_M and its six truncated mutants incubated in 10 mM NaH _2_PO _4_-H _3_PO _4_ (pH 2.5), diluted into 20 mM NaH _2_PO _4_-Na _2_HPO _4_ buffer (pH 7.4), and incubated for 1 h at 25°C with different concentrations of GdnSCN (0-3.5 M) or different concentrations of GdnHCl (0–6.5 M), giving a final total volume of 60 μL and a final concentration of 17 μM for β _2_M fibrils. The concentration of GdnSCN or GdnHCl was then adjusted to 0.35 or 0.65 M, followed by a ThT binding assay. A Cytation 3 Cell Imaging Multi-Mode Reader (BioTek, Winooski, USA) was used to measure ThT fluorescence produced, with excitation at 450 nm and emission at 480 nm. The half-concentration of GdnSCN or GdnHCl at which the ThT fluorescence intensity of β _2_M fibrils is decreased by 50% ( *C*_1⁄2_) of the ΔN6 fibrils and WT β _2_M fibrils was determined using a sigmoidal equation [ 25, 40– 42] using the above ThT fluorescence data.

### CD spectroscopy

Far-UV CD measurements were performed by using a ChirascanTM V100 spectrometer (Applied Photophysics, Surrey, UK) with bandwidth of 1 nm, time-per-point of 0.8 s and step size of 1 nm. Quartz cell with a 0.5 mm light-path was used for measurements and spectra were recorded from 190 nm to 260 nm. One hundred microliters of β _2_M fibril samples were diluted into 10 mM NaH _2_PO _4_-H _3_PO _4_ (pH 2.5), giving a final volume of 0.85 mL and a final concentration of 10 μM for β _2_M fibrils. All scanned spectra were corrected relative to buffer blank. The mean residue molar ellipticity [ *θ*] (deg∙ cm ^2^∙ dmol ^-1^) was calculated using the formula: [ *θ*]=( *θ*_obs_/10)(MRW/ *lc*) as described by Liang lab [25​], where *θ*_obs_ is the observed ellipticity in deg, MRW is the mean residue molecular weight (118.6 Da for WT β _2_M, ΔN2, and ΔN5, 118.7 Da for ΔN1, 118.2 Da for ΔN3, 118.4 Da for ΔN4, and 118.5 Da for ΔN6), *l* is the path length in cm, and *c* is the protein concentration in g/mL. Measurements were performed at 25 °C.

### Dynamics multi-mode spectroscopy

Thermal stability assays of β _2_M fibrils were performed on the ChirascanTM V100 Spectrometer (Applied Photophysics) with a dynamics multi-mode spectroscopy. The fibril samples were diluted in 10 mM NaH _2_PO _4_-H _3_PO _4_ (pH 2.5) with a final concentration of 25 μM and a final total volume of 160 μL. Quartz cell with a 0.5 mm light-path was used for measurements and spectra recorded from 190 nm to 260 nm were collected with bandwidth of 1 nm, step size of 1 nm, time-per-point of 0.8 s, temperature range of 25°C to 95°C, temperature rate of 1°C/min, and temperature step of 2°C. All scanned spectra were corrected relative to buffer blank. The data was analyzed by using the Global 3 Analysis Software (Applied Photophysics) with a sigmoidal equation described above to obtain the parameters.

### Congo red binding assays

β _2_M fibrils were analyzed by Congo red binding assays. A stock solution of 1 mM Congo red was prepared in 10 mM NaH _2_PO _4_-Na _2_HPO _4_ buffer (pH 7.4) and filtered through a filter of 0.22 μm pore size before use. In a typical assay, the β _2_M fibril sample was mixed with a solution of Congo red to yield a final Congo red concentration of 50 μM and a final β _2_M concentration of 10 μM, and the absorbance spectrum between 400 and 700 nm was then recorded on a Cytation 3 Cell Imaging Multi-Mode Reader (BioTek). Measurements were performed at 25°C.

### Atomic force microscopy

A total of 100 μL of β _2_M fibrils prepared in 10 mM NaH _2_PO _4_-H _3_PO _4_ (pH 2.5) were diluted into H _2_O, giving a final volume of 0.50 mL. Ten microliters of β _2_M fibril samples (~17 μM) were then incubated on a freshly cleaved mica surface for 5 min, followed by rinsing three times with 10 μL of pure water to remove the unbound fibrils and drying at room temperature. The fibrils on the mica surface were probed in air by the Dimension icon scanning probe microscope (Bruker, Santa Barbara, USA) with ScanAsyst mode. The measurements were realized by using SCANASYST-AIR probe with a spring constant of 0.4 N/m and a resonance frequency of 70 kHz (Bruker). A fixed resolution (256×256 data points) of the AFM images was acquired with a scan rate at 1 Hz and analyzed by using NanoScope Analysis 2.0 software (Bruker).

### Transmission electron microscopy

β _2_M fibrils were examined by transmission electron microscopy of negatively stained samples. Ten microliters of β _2_M fibril samples (~17 μM) were loaded on 300 mesh copper grids for 1–2 min and washed with H _2_O for 10 s. Samples on grids were then stained with 2% (w/v) uranyl acetate for 5 s and dried in air at 25°C. The stained samples were examined using a JEM-1400 Plus transmission electron microscope (JEOL, Tokyo, Japan) operating at 100 kV.

### Sarcosyl-insoluble SDS-PAGE

During seed-dependent experiments, a series of samples (60 μL) were taken in chronological order corresponding to ThT fluorescence assays. Sixty microliters of 2% sarcosyl solution were added into the samples and incubated for 30 min at 37°C. Then the samples were centrifuged for 45 min at 150,000 *g* and 4°C. The pellets were further washed with 20 mM NaH _2_PO _4_-Na _2_HPO _4_ buffer (pH 7.4) and centrifuged at 150,000 *g* and 4°C for 45 min. The resulting pellets were mixed with 5×loading buffer and separated by 15% SDS-PAGE. The insoluble β _2_M fibrils were detected by SDS-PAGE with Coomassie Blue R250 staining.

### Statistical analysis

The data shown for each experiment were based on at least three technical replicates, as indicated in individual figure legends. Data are presented as the mean±SD, and *P-*values were determined using the Student’s *t* test. All experiments were further confirmed by biological repeats.

## Results

### The ΔN6 β _2_M fibril exhibits a significantly lower conformational stability than the wild-type at acidic pH 2.5

**Figure 1:**
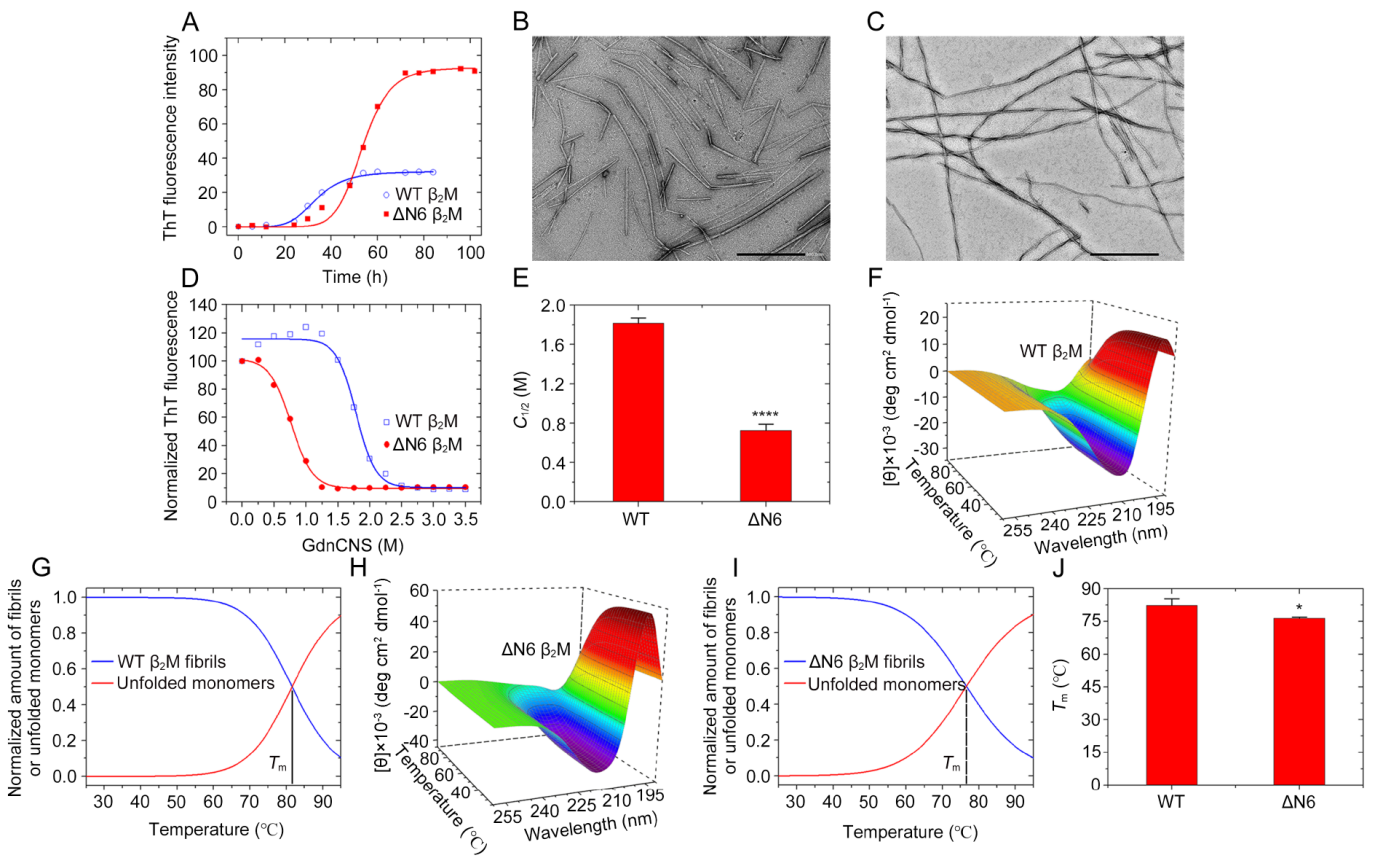
Figure1 **ΔN6 β _2_M fibrils exhibite a significantly lower conformational stability than WT β _2_M fibrils at acidic pH 2.5 **(A) Samples (85 μM) of WT β2M (blue) and ΔN6 β2M (red) were incubated at pH 2.5 with agitation at 220 rpm and 37°C. The solid lines show the best sigmoidal fit for the ThT intensity-time curves. (B, C) TEM images of amyloid fibrils formed by WT β2M (B) incubated for 84 h and ΔN6 β2M (C) incubated for 102 h at pH 2.5. Scale bar: 500 nm. (D) GdnCNS concentration-dependent denaturation profiles monitored by ThT fluorescence for amyloid fibrils produced from WT β2M (blue) and ΔN6 β2M (red) at pH 2.5, which were incubated for 1 h at 25°C with increasing concentrations of GdnCNS. (E) The C1/2 values for amyloid fibrils of WT β2M and ΔN6 β2M were determined using a sigmoidal equation and are expressed as the mean±SD of the values obtained from 3 independent experiments. C1/2, P = 0.038. (F-I) Three-dimensional (3D) diagrams of the CD spectra against temperature ranged from 25°C to 95°C for amyloid fibrils (25 μM) of WT β2M (F) and ΔN6 β2M (H) formed at pH 2.5; the normalized amount of the fibrils (blue) and the unfolded monomers (red) for WT β2M (G) and ΔN6 β2M (I). The isosbestic points represent the corresponding Tm values. (J) The Tm values were also determined using a sigmoidal equation and are expressed as the mean±SD of the values obtained from 3 independent experiments. Tm, P= 0.000021. The Student’s t test was used to perform statistical analyses. Values of P<0.05 indicate statistically significant differences. *P<0.05, and ****P<0.0001 relative to WT β2M (a control).

One of the pathological features of dialysis-associated amyloidosis is the formation of amyloid fibrils consisting of WT β _2_M and its truncated variant ΔN6 in the joint spaces [ 10, 12, 13, 28, 30]. In order to clarify the role of the first six amino acids in β _2_M’s fibril formation, we performed experiments at acidic pH 2.5 to explore fibril formation of this protein. Our results showed that WT β _2_M and ΔN6 exhibited a significant increase in ThT intensity over the timescale ( Figure 1A), indicating that ΔN6 β _2_M and its wild-type did form fibrils at acidic pH 2.5, as previously reported [ 25, 29, 30, 35]. Negative-staining TEM imaging showed that under such conditions, WT β _2_M mainly formed straight filaments ( Figure 1B) but ΔN6 β _2_M mainly formed twisted fibrils (i.e. two protofilaments intertwined into a helix) ( Figure 1C). Thus, ΔN6 β _2_M formed fibrils with a morphology distinct from WT β _2_M at acidic pH 2.5.

We next examined whether the ΔN6 β _2_M fibril exhibits distinct conformational stability from the WT β _2_M fibril at acidic pH 2.5. Chemical and/or thermal denaturation was widely used to evaluate the conformational stability of amyloid fibrils [ 35, 40]. GdnHCl, a commonly used denaturing agent for protein, was employed in our denaturation assay ( Supplementary Figure S2A). The *C*_1⁄2_ value of the WT β _2_M fibril incubated with GdnHCl is 4.046±0.062 M ( Supplementary Fig S2B). Notably, the *C*_1⁄2_ value of the ΔN6 β _2_M fibril is 2.639±0.039 M ( Supplementary Figure S2B), which is significantly lower than that of the WT fibril, suggesting that the ΔN6 truncation significantly decreased the conformational stability of β _2_M fibrils. A strong chaotropic salt, GdnSCN, which is a stronger denaturing agent than GdnHCl, was used in our denaturation assay ( Figure 1D). The *C*_1⁄2_ value of the WT β _2_M fibril incubated with GdnSCN is 1.820±0.051 M ( Figure 1E). Notably, the *C*_1⁄2_ value of the ΔN6 β _2_M fibril is 0.726±0.064 M ( Figure 1E), which is significantly lower than that of the WT fibril, suggesting that the ΔN6 β _2_M fibril is less stable than the wild-type at acidic pH 2.5. Intriguingly, Esposito *et al*. [30] did a comprehensive structural and functional analysis of ΔN6 β _2_M; they measured the unfolding of the protein induced by GdnHCl and determined *C*_1⁄2_ values of 2.23 M and 1.65 M for the unfolding of wild-type and ΔN6 β _2_M, respectively, which are substantially lower than the values for the WT β _2_M fibril and the ΔN6 fibril incubated with GdnHCl reported in this study.

To validate the stability differences between ΔN6 β _2_M and WT fibrils, we further measured their thermostabilities. The β _2_M fibrils were incubated with 10 mM NaH _2_PO _4_-H _3_PO _4_ (pH 2.5) under a thermal gradient from 25 to 95°C, and the unfolded monomers disassembled from β _2_M fibrils were measured by dynamic multi-mode spectroscopy ( Figure 1F–I) and distinguished by far-UV CD spectroscopy ( Supplementary Figure S3A–C). The unfolded monomers at 95°C were characterized by a largely random coil structure with a strong negative peak at 203 nm ( Supplementary Figure S3A), which is similar to those induced by acid at pH 2.5 ( Supplementary Figure S3B). In sharp contrast, the β _2_M fibrils at 25°C were characterized by β-sheet-rich structures with a negative peak at 216 nm ( Supplementary Figure S3C). 3D far-UV CD spectra for fibrils of WT β _2_M and ΔN6 are shown in Figure 1F,H. The results showed that the melting temperature ( *T*_m_) value of ΔN6 β _2_M fibrils is 76.5±0.5°C, significantly lower than that of WT β _2_M fibrils (82.3±3.3°C) ( Figure 1G,I,J). Together, these results demonstrated that the ΔN6 β _2_M fibril has a significantly lower conformational stability compared to the WT fibril at acidic pH 2.5. Therefore, the loss of the first six residues alters the stability of the overall fold of β _2_M [30] and significantly decreases the conformational stability of β _2_M fibrils (this work).

### The residues 4 to 6 at the N-terminus in particular modulate amyloid fibril formation of β _2_M under more physiological conditions (pH 6.2)

**Figure 2:**
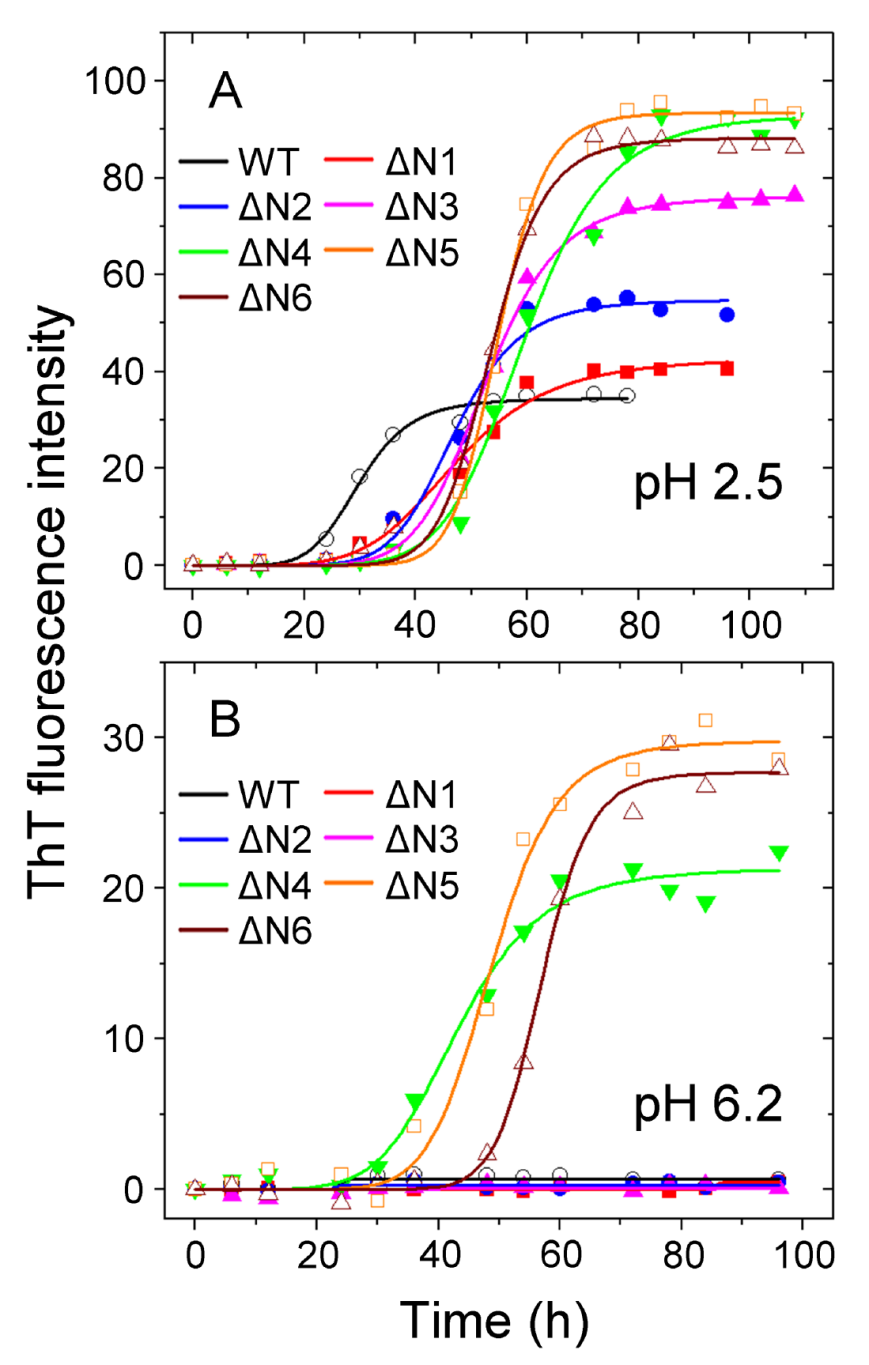
Figure2 **Modulation of amyloid fibril formation of β _2_M by residues 4 to 6 at the N-terminus under more physiological conditions (pH 6.2) analyzed by ThT binding assays **(A,B) Amyloid fibril formation of WT β2M and its six truncated variants at pH 6.2 or 2.5. Samples (85 μM) of WT β2M (black circle) and its truncated mutants ΔN1 (red square), ΔN2 (blue circle), ΔN3 (magenta triangle), ΔN4 (green inverse triangle), ΔN5 (orange square), and ΔN6 (wine triangle) were incubated at either pH 2.5 (A) or pH 6.2 (B) with agitation at 220 rpm and 37°C. The solid lines show the best sigmoidal fit for the ThT intensity-time curves. At acidic pH 2.5, we produced amyloid fibrils from WT β2M and its six truncated variants. At more physiological pH 6.2, however, we assembled amyloid fibrils only from ΔN4, ΔN5, and ΔN6 β2M but not from WT β2M and its three truncated variants ΔN1 to ΔN3. All ThT binding assays were repeated at least three times, and the results were reproducible.

**Figure 3:**
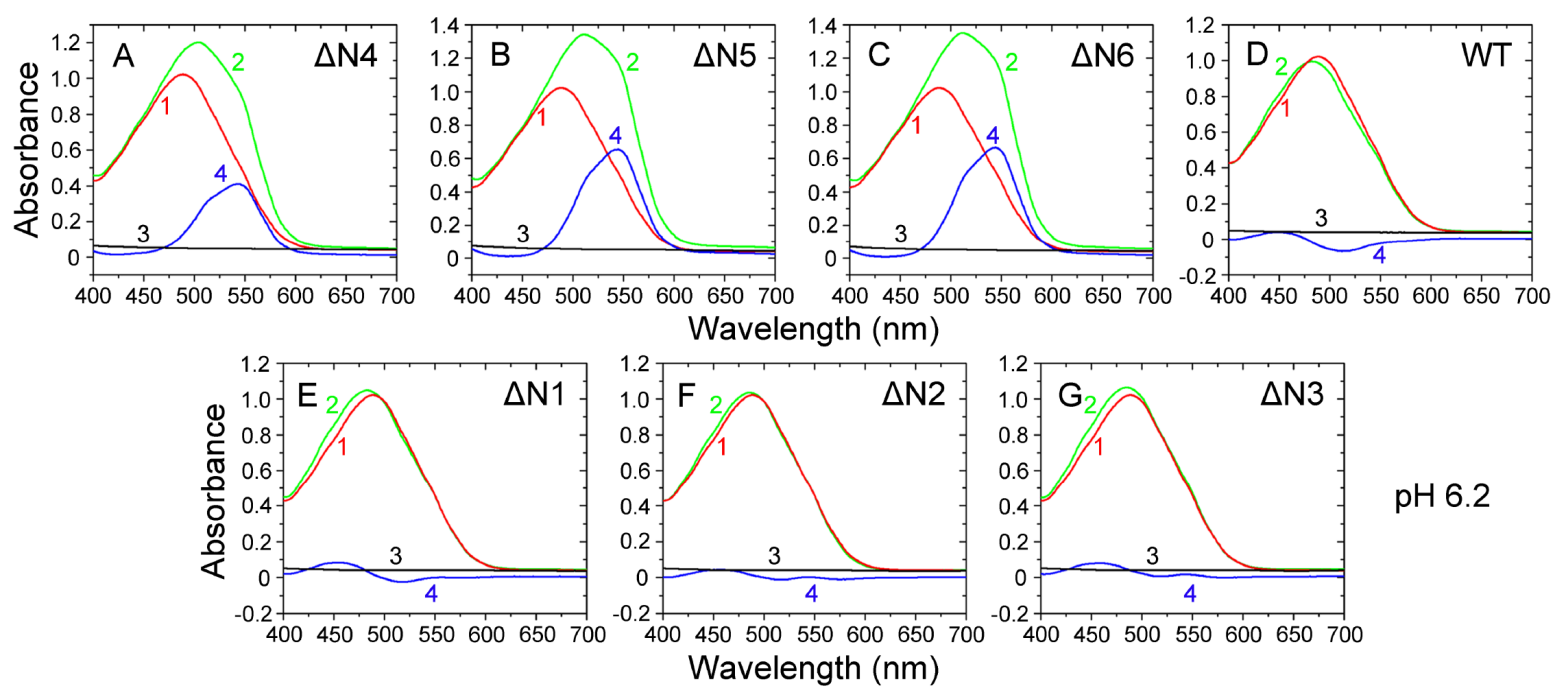
Figure3 **Modulation of amyloid fibril formation of β _2_M by residues 4 to 6 at the N-terminus under more physiological conditions (pH 6.2) analyzed by Congo red binding assays **Amyloid fibrils of WT β2M and its N-terminally-truncated variants were formed at pH 6.2 with agitation at 220 rpm and 37°C. Absorbance data are shown for amyloid fibrils at the end of fibril formation for 10 μM WT β2M (D) and its truncated mutants ΔN1 (E), ΔN2 (F), ΔN3 (G), ΔN4 (A), ΔN5 (B), and ΔN6 (C) in the presence of 50 μM Congo red at 25°C. The difference spectra (Curve 4, blue) with the maximum absorbance at 550 nm were obtained by subtracting the absorbance spectra of β2M fibrils alone (Curve 3, black) and Congo red alone (Curve 1, red) with the maximum absorbance at 490 nm from those of β2M fibrils + Congo red (Curve 2, green). At pH 6.2, we assembled amyloid fibrils only from ΔN4, ΔN5, and ΔN6 β2M but not from WT β2M and its three truncated variants ΔN1 to ΔN3. All Congo red binding assays were repeated at least three times, and the results were reproducible.

**Figure 4:**
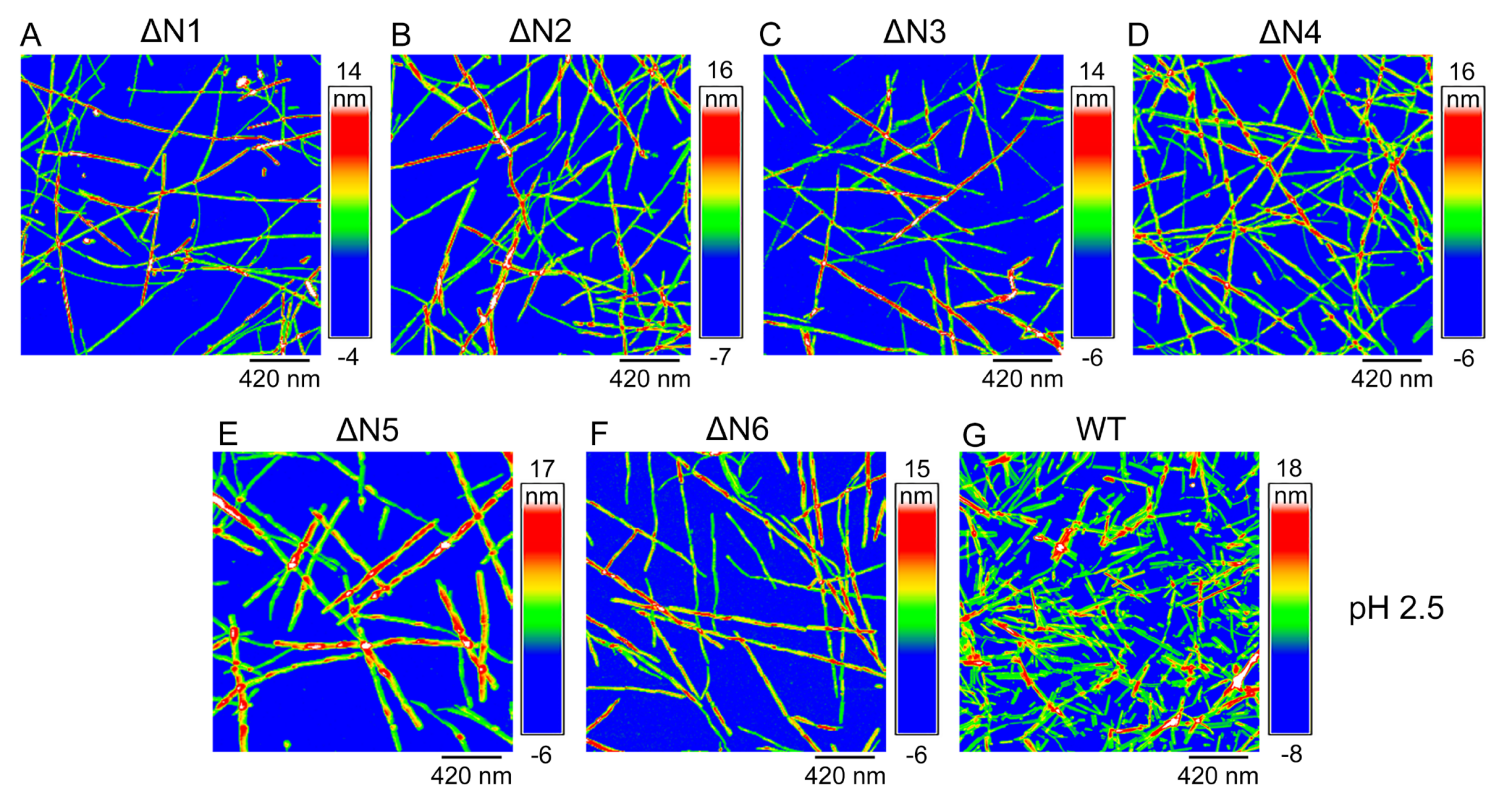
Figure4 **High-resolution AFM images of β _2_M fibrils at acidic pH 2.5 **AFM images of amyloid fibrils formed by WT β2M incubated for 78 h (G), ΔN1 (A), and ΔN2 (B) incubated for 96 h, and ΔN3 (C), ΔN4 (D), ΔN5 (E), and ΔN6 (F) incubated for 108 h at pH 2.5 with agitation at 220 rpm and 37°C. The three different time points (78, 96, and 108 h) were used because the incubation times at the end of fibril formation for WT β2M and ΔN1 to ΔN6 were different. Scale bar: 420 nm. At pH 2.5, we produced amyloid fibrils from WT β2M and its six truncated variants.

**Figure 5:**
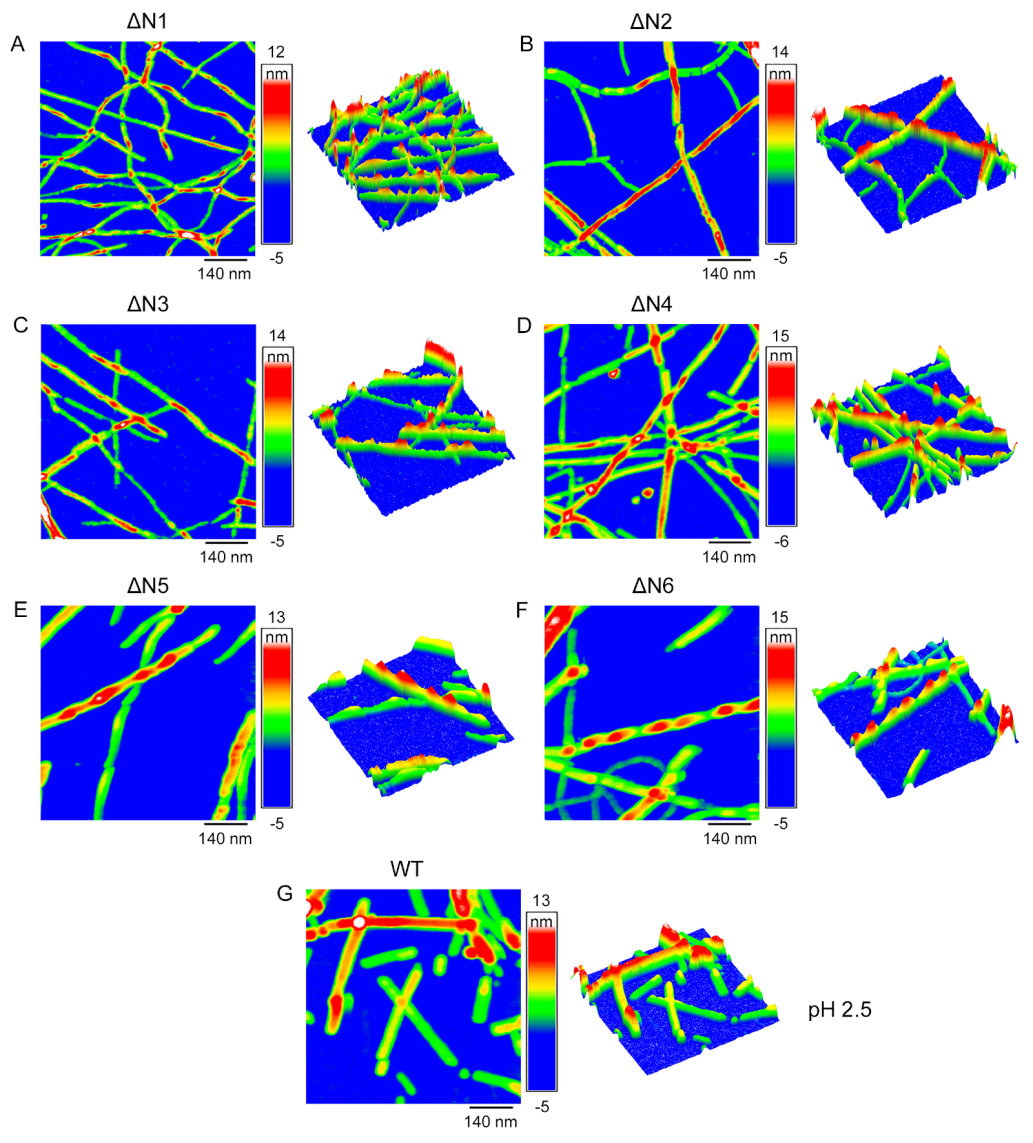
Figure5 **High-resolution 3D AFM images of β _2_M fibrils at acidic pH 2.5 **2D (left) and 3D (right) AFM images of amyloid fibrils formed by WT β2M incubated for 78 h (A), ΔN1 (B) and ΔN2 (C) incubated for 96 h, and ΔN3 (D), ΔN4 (E), ΔN5 (F), and ΔN6 (G) incubated for 108 h at pH 2.5 with agitation at 220 rpm and 37°C. Scale bar: 140 nm.

As mentioned below, an extensive experimental examination was conducted into the role of the first six residues of β _2_M in modulating its amyloid fibril formation (Figures 2– 5 and Supplementary Figure S4, S5). Six mutants lacking up to six residues at the N-terminus, namely ΔN1 lacking ^1^I, ΔN2 lacking ^1^IQ ^2^, ΔN3 lacking ^1^IQR ^3^, ΔN4 lacking four residues at the N-terminus, ΔN5 lacking ^1^IQRTP ^5^, and ΔN6 lacking six residues at the N-terminus, were used. Congo red, an anionic dye, was used to selectively stain amyloid fibrils [ 44– 46]. Our results showed that at acidic pH 2.5, WT β _2_M and its six truncated variants (from ΔN1 to ΔN6) exhibited a significant increase in ThT intensity over the timescale ( Figure 2A), indicating that ΔN1–ΔN6 and their wild-type did form fibrils under such conditions. In other words, at acidic pH 2.5, amyloid fibrils were produced *in vitro* from recombinant, WT β _2_M and its six truncated variants ( Figure 2A). At more physiological pH (pH 6.2), however, amyloid fibrils were assembled *in vitro* only from recombinant, ΔN4, ΔN5, and ΔN6 β _2_M but not from WT β _2_M and its three truncated variants ΔN1 to ΔN3, demonstrated by the ThT binding assays ( Figure 2B). These data clearly demonstrated that the residues 4 to 6 (TPK) at the N-terminus in particular modulated amyloid fibril formation of β _2_M under more physiological conditions (pH 6.2), which was confirmed by our Congo red binding assays. Congo red binding assays showed a red shift of the maximum absorbance, from 490 nm (red peak) to 550 nm (blue peak), in the presence of ΔN4 fibrils ( Figure 3A), ΔN5 fibrils ( Figure 3B) or ΔN6 fibrils ( Figure 3C) at more physiological pH 6.2, which is typical of amyloid fibrils [ 44– 46]. However, we did not observe the blue peak at 550 nm for WT β _2_M ( Figure 3D) and its three truncated variants ΔN1 to ΔN3 ( Figure 3E–G) at pH 6.2, indicating that ΔN1–ΔN3 and their wild-type did not form amyloid fibrils under such conditions. Thus, removal of the first six, five or four residues led to enhanced fibril formation of β _2_M at more physiological pH 6.2. Congo red binding assays also showed a red shift of the maximum absorbance, from 490 to 550 nm, in the presence of WT β _2_M fibrils ( Supplementary Figure S4A) or ΔN1–ΔN6 fibrils ( Supplementary Figure S4B–G) at acidic pH 2.5, indicating that ΔN1–ΔN6 and their wild-type did form amyloid fibrils under such conditions.

We next examined whether the ΔN1–ΔN5 fibrils exhibit distinct conformational stability from the WT β _2_M fibril at acidic pH 2.5. GdnSCN was also used in our denaturation assay ( Supplementary Figure S6A–E). The *C*_1⁄2_ value of the ΔN1 fibril, the ΔN2 fibril, and the ΔN3 fibril incubated with GdnSCN is 1.676±0.004 M, 1.557±0.033 M, and 1.486±0.060 M, respectively ( Supplementary Figure S6F), which is significantly lower than that of the WT fibril, suggesting that the ΔN1–ΔN3 fibrils are less stable than the wild-type at acidic pH 2.5. Notably, the *C*_1⁄2_ value of the ΔN4 β _2_M fibril, the ΔN5 fibril, and the ΔN6 fibril incubated with GdnSCN is 0.940±0.022 M, 0.777±0.024 M ( Supplementary Figure S6F), and 0.726±0.064 M ( Figure 1E), respectively, which is much significantly lower than that of the WT fibril, suggesting that the ΔN6 to ΔN4 truncation decreased the conformational stability of β _2_M fibrils more significantly than the ΔN3 to ΔN1 truncation.

### High-resolution AFM and TEM images of β _2_M fibrils at acidic pH 2.5

High-resolution AFM and TEM are powerful tools to visualize the morphology of amyloid fibrils [ 41, 46– 48]. The heterogeneity of amyloid fibrils can be quantitatively assessed using AFM images by analyzing the variations in the fibrils’ mesoscopic arrangements such as fibril width, fibril length, and twist pitch [ 46– 48]. High-resolution AFM imaging and TEM imaging showed that ΔN1, ΔN2, ΔN3, ΔN4, ΔN5, and ΔN6 formed similar unbranched fibrils and mainly produced twisted fibrils (i.e. two protofilaments intertwined into a helix), with a fibril width of 15.2±1.3 nm, 15.2±1.6 nm, 15.1±1.6 nm, 15.1±1.3 nm, 15.3±1.7 nm, and 15.4±1.6 nm ( *n*=50), respectively, and a fibril length of 1765±964 nm, 1832±826 nm, 1896±783 nm, 1902±836 nm, 2056±977 nm, and 2070±1020 nm ( *n*=100), respectively ( Figure 4A–F, Figure 5A–F **,** and Supplementary Figure S5A–F). In comparison, WT β _2_M mainly formed straight filaments with a fibril width of 15.3±1.4 nm ( *n*=50), which is similar to those of ΔN1 to ΔN6, and a fibril length of 902±591 nm ( *n*=100) ( Figures 4G,5G, and Supplementary Figure S5G), which is substantially shorter than those of ΔN1 to ΔN6. Together, these results demonstrated that the loss of the first six residues possibly alters the morphology of β _2_M fibrils.

### Homo- and heterotypic seeding of ΔN6 fibrils strongly promotes amyloid fibril formation of WT β _2_M and its six truncated variants at acidic pH 2.5

**Figure 6:**
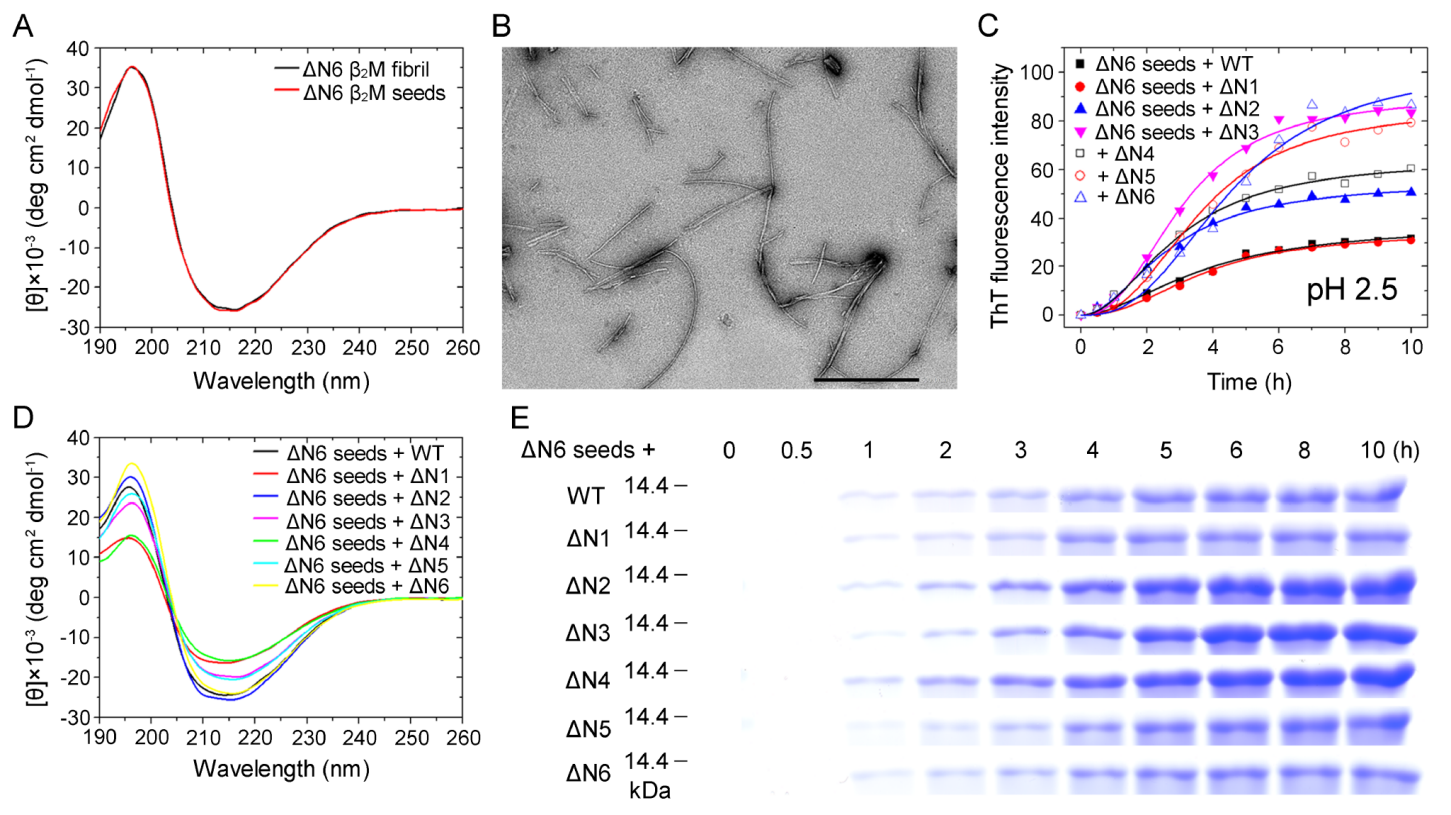
Figure6 **Homo- and heterotypic seeding of ΔN6 fibrils strongly promoted amyloid fibril formation of WT β _2_M and its six truncated variants at acidic pH 2.5 **(A) The secondary structures of ΔN6 fibrils (black) and ΔN6 fibril seeds (red) at pH 2.5 monitored by far-UV CD. (B) TEM images of ΔN6 fibril seeds at pH 2.5. Scale bar: 500 nm. (C) Samples (85 μM) of WT β2M (solid square) and its six truncated mutants ΔN1 (solid circle), ΔN2 (solid up triangle), ΔN3 (solid inverse triangle), ΔN4 (open square), ΔN5 (open circle), and ΔN6 (open triangle) in the presence of 2% (v/v) ΔN6 fibril seeds were incubated at pH 2.5 with agitation at 220 rpm and 37°C. The solid lines show the best fit for the ThT intensity-time curves. (D) CD spectra at the end of fibril formation (10 h) for WT β2M (black) and its six truncated variants ΔN1 (red), ΔN2 (blue), ΔN3 (magenta), ΔN4 (green), ΔN5 (cyan), and ΔN6 (yellow). (E) SDS-PAGE analysis of time-dependent sarkosyl-insoluble β2M incubated with 2% (v/v) ΔN6 fibril seeds at pH 2.5, including WT β2M and its six truncated mutants. 85 μM β2M samples were incubated with 2% sarkosyl and separated by 15% SDS-PAGE. The insoluble β2M monomers were detected by Coomassie Blue R250 staining.

We next analyzed homo- and heterotypic seeding of amyloid fibrils of WT human β _2_M and its N-terminally-truncated variants ΔN1 to ΔN6, lacking up to six residues at the N-terminus, at acidic pH 2.5. Seed-dependent propagation experiments were carried out in the presence of 2% (v/v) ΔN6 fibril seeds at pH 2.5 ( Figure 6 and Supplementary Figures S7, S8). Structural features of ΔN6 fibril seeds were characterized by far-UV CD and TEM ( Figure 6A,B). The CD spectra of the seeds well matched with those of the ΔN6 fibril ( Figure 6A), indicating that both are a β-sheet-rich architecture at pH 2.5. Then, TEM ( Figure 6B) and seeding experiments ( Figure 6C) were used to judge the successful preparation of the ΔN6 fibril seed. Here “seed” is a technical term. Negative-staining TEM imaging showed that the seeds did contain short fibrils and protofilaments under such conditions ( Figure 6B). More importantly, the addition of the seeds significantly reduced the lag time of fibrillization of recombinant, WT β _2_M and its six truncated variants ( Figure 6C). As a mild anionic surfactant, sarcosyl is able to dissolve the monomers and oligomers of proteins rather than amyloid fibrils and has been used to semi-quantify the aggregates of proteins [41]. Notably, we showed that when incubated with the ΔN6 fibril seeds, WT β _2_M and its six truncated variants ΔN1–ΔN6 all exhibited a significant increase in ThT intensity over the timescale ( Figure 6C), a strong negative peak at 216 nm ( Figure 6D), and a clear band corresponding to sarcosyl-insoluble β _2_M fibrils (for 1–10 h) ( Figure 6E), indicating that ΔN1-ΔN6 and their wild-type did form fibrils induced by the ΔN6 fibril seeds at acidic pH 2.5. Congo red binding assays also showed a red shift of the maximum absorbance, from 490 to 550 nm, in the presence of ΔN6 fibril seed-induced WT β _2_M fibrils ( Supplementary Figure S7A) or ΔN6 fibril seed-induced ΔN1–ΔN6 fibrils at pH 2.5 ( Supplementary Figure S7B–G), which is typical of amyloid fibrils [ 44– 46]. Negative-staining TEM imaging showed that in the presence of the ΔN6 fibril seeds, ΔN1–ΔN6 and their wild-type all formed unbranched amyloid fibrils and mainly produced twisted fibrils after incubation for 10 h under such conditions ( Supplementary Figure S8A–G), similar to the morphology of ΔN6 fibrils ( Supplementary Figure S5F). Together, these results demonstrated that homo- and heterotypic seeding of ΔN6 fibrils strongly promotes amyloid fibril formation of WT β _2_M and its six truncated variants at acidic pH 2.5.

### Homo- and heterotypic seeding of WT β _2_M fibrils facilitates amyloid fibril formation of WT β _2_M and its three truncated variants ΔN1 to ΔN3 but not ΔN4 to ΔN6 at acidic pH 2.5

**Figure 7:**
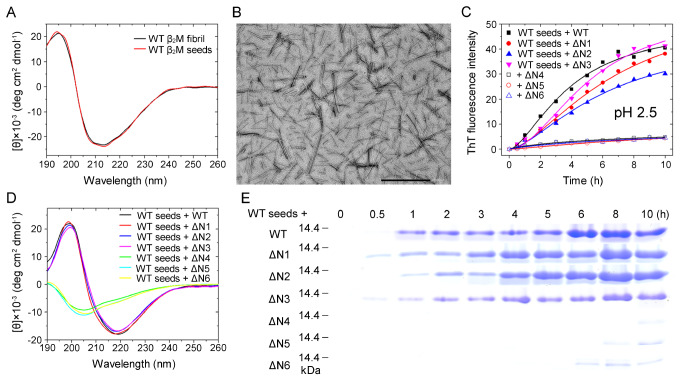
Figure7 **Homo- and heterotypic seeding of WT β _2_M fibrils facilitated amyloid fibril formation of the wild-type protein and its three truncated variants ΔN1 to ΔN3 but not ΔN4, ΔN5, and ΔN6 at acidic pH 2.5 **(A) The secondary structures of WT β2M fibrils (black) and WT β2M fibril seeds (red) at pH 2.5 monitored by far-UV CD. (B) TEM images of WT β2M fibril seeds at pH 2.5. Scale bar: 500 nm. (C) Samples (85 μM) of WT β2M (solid square) and its six truncated mutants ΔN1 (solid circle), ΔN2 (solid up triangle), ΔN3 (solid inverse triangle), ΔN4 (open square), ΔN5 (open circle), and ΔN6 (open triangle) in the presence of 2% (v/v) WT β2M fibril seeds were incubated at pH 2.5 with agitation at 220 rpm and 37°C. The solid lines show the best fit for the ThT intensity-time curves. (D) CD spectra at the end of fibril formation (10 h) for WT β2M (black) and its six truncated variants ΔN1 (red), ΔN2 (blue), ΔN3 (magenta), ΔN4 (green), ΔN5 (cyan), and ΔN6 (yellow). (E) SDS-PAGE analysis of time-dependent sarkosyl-insoluble β2M incubated with 2% (v/v) WT β2M fibril seeds at pH 2.5, including WT β2M and its six truncated mutants.

We have shown that ΔN6 fibril seeds have a strong ability to propagate amyloid formation of WT β _2_M and its six truncated variants ΔN1–ΔN6 at acidic pH 2.5. We then carried out seed-dependent propagation experiments in the presence of 2% (v/v) WT β _2_M fibril seeds at acidic pH 2.5 ( Figure 7 and Supplementary Figure S9). Structural features of WT β _2_M fibril seeds were characterized by far-UV CD and TEM ( Figure 7A,B). The CD spectra of the seeds well matched with those of the WT β _2_M fibril ( Figure 7A), indicating that both are a β-sheet-rich architecture at pH 2.5. TEM ( Figure 7B) and seeding experiments ( Figure 7C) were further employed to judge the successful preparation of the WT β _2_M fibril seed. Negative-staining TEM imaging showed that the seeds did consist of short fibrils and protofilaments under such conditions ( Figure 7B). More importantly, the addition of the seeds significantly reduced the lag time of fibrillization of recombinant, WT β _2_M and its three truncated variants ΔN1–ΔN3 ( Figure 7C). Notably, we showed that when incubated with the WT β _2_M fibril seeds, WT β _2_M and its three truncated variants ΔN1–ΔN3 exhibited a significant increase in ThT intensity over the timescale ( Figure 7C), a strong negative peak at 218 nm ( Figure 7D), and a clear band corresponding to sarcosyl-insoluble β _2_M fibrils (for 1–10 h) ( Figure 7E), indicating that ΔN1–ΔN3 and their wild-type did form fibrils induced by the WT fibril seeds at acidic pH 2.5. Congo red binding assays showed a red shift of the maximum absorbance, from 490 to 550 nm, in the presence of WT β _2_M fibrils ( Supplementary Figure S9A) or ΔN1–ΔN3 fibrils ( Supplementary Figure S9B–D) all induced by the WT fibril seeds at pH 2.5, which is typical of amyloid fibrils [ 44– 46]. In sharp contrast, the addition of the WT fibril seeds into ΔN4–ΔN6 monomers did not effectively induce the formation of ΔN4–ΔN6 fibrils at acidic pH 2.5 ( Figure 7C–E and Supplementary Figure S9E–G). Thus, WT β _2_M fibril seeds have a strong ability to propagate fibril formation of WT β _2_M and its truncated variants ΔN1–ΔN3, but only have a very weak ability to propagate fibril formation of ΔN4–ΔN6 under such conditions. The CD signal of the samples that apparently show low seeding activity is surprisingly weak ( Figure 7D; WT β _2_M fibril seeds + ΔN4–ΔN6). The interpretation that seeding is weak would suggest that the ΔN4–ΔN6 monomers retain the acid-denatured conformation of β _2_M. This is visible as a random coil signal with a molar ellipticity at 203 nm of –10 deg∙ cm ^2^∙ dmol ^-1^ (this work and [49]). Together, these results demonstrated that at acidic pH 2.5, homo- and heterotypic seeding of WT β _2_M fibrils facilitates amyloid fibril formation of WT β _2_M and its three truncated variants ΔN1 to ΔN3 but not ΔN4 to ΔN6, indicating that the residues 4 to 6 (TPK) at the N-terminus in particular modulate amyloid fibril formation of β _2_M under such conditions.

### Homo- and heterotypic seeding of ΔN6 fibrils strongly promotes amyloid fibril formation of WT β _2_M and its six truncated variants at more physiological pH 6.2

**Figure 8:**
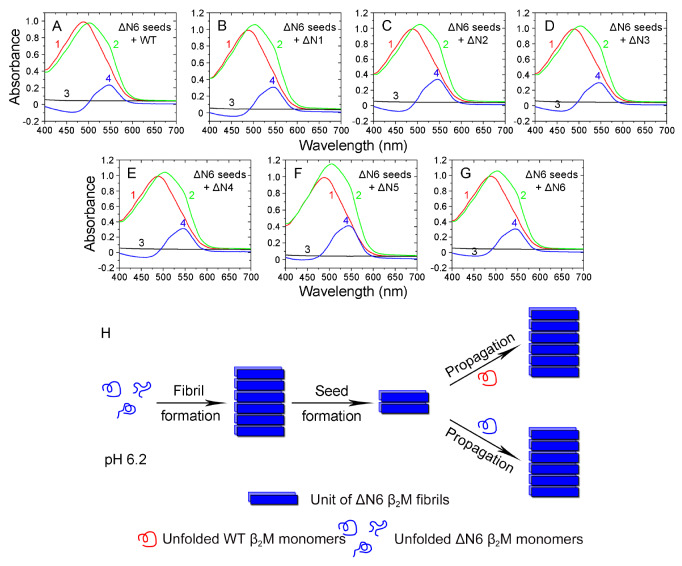
Figure8 **Homo- and heterotypic seeding of ΔN6 fibrils strongly promoted amyloid fibril formation of WT β _2_M and its six truncated variants at more physiological conditions (pH 6.2) **(A–G) Amyloid fibrils of WT β2M and its N-terminally-truncated variants were induced by 10% (v/v) ΔN6 fibril seeds at pH 6.2 with agitation at 220 rpm and 37°C. Absorbance data are shown for amyloid fibrils at the end of fibril formation for 10 μM WT β2M (A) and its truncated mutants ΔN1 (B), ΔN2 (C), ΔN3 (D), ΔN4 (E), ΔN5 (F), and ΔN6 (G) in the presence of 50 μM Congo red at 25°C. The difference spectra (Curve 4, blue) with the maximum absorbance at 550 nm were obtained by subtracting the absorbance spectra of β2M fibrils alone (Curve 3, black) and Congo red alone (Curve 1, red) with the maximum absorbance at 490 nm from those of β2M fibrils + Congo red (Curve 2, green). All Congo red binding assays were repeated at least three times, and the results were reproducible. (H) A hypothetical model showing how the six residues at the N-terminus modulate fibril propagation of β2M and the interactions of WT β2M with N-terminally truncated variants at more physiological pH 6.2. Under such conditions, the loss of the first six residues (ΔN6) facilitates amyloid fibril (blue brick) formation from ΔN6 β2M monomers (blue rope), and homo- and heterotypic seeding of ΔN6 fibrils strongly promotes amyloid fibril formation of WT β2M (red rope) and its truncated variant ΔN6 (blue rope).

We have shown that ΔN6 fibril seeds have a strong ability to propagate amyloid formation of WT β _2_M and its six truncated variants ΔN1–ΔN6 at acidic pH 2.5. We then analyzed homo- and heterotypic seeding of ΔN6 β _2_M under more physiological conditions (pH 6.2). Seed-dependent propagation experiments were carried out in the presence of 10% (v/v) ΔN6 fibril seeds at pH 6.2 ( Figure 8). Congo red binding assays showed a red shift of the maximum absorbance, from 490 to 550 nm, in the presence of ΔN6 fibril seed-induced WT β _2_M fibrils ( Figure 8A) or ΔN6 fibril seed-induced ΔN1–ΔN6 fibrils at pH 6.2 ( Figure 8B–G), which is typical of amyloid fibrils [ 44– 46]. Together, these results demonstrated that homo- and heterotypic seeding of ΔN6 fibrils strongly promotes amyloid fibril formation of WT β _2_M and its six truncated variants at more physiological pH 6.2.

## Discussion

Various *in vitro* assays have confirmed that N-terminal hexapeptide has an impact on the fibrillization of β _2_M [ 29, 30, 33, 35– 37], but the mechanisms behind the phenomenon are still not clear. ΔN6, a pathological mutant of β _2_M lacking the N-terminal six amino acids, is more prone to aggregation and capable of converting WT β _2_M into an aggregation competent conformer [ 29, 50]. In the present study, we explored the roles of the first six residues at the N-terminus of β _2_M in the interactions and the modulation of amyloid fibril propagation of β _2_M. Accumulating evidence pointed to a crucial role of ΔN6 in dialysis-related amyloidosis associated with β _2_M aggregation and deposition [ 28– 30]. Cross-seeding of WT β _2_M and ΔN6 has been studied at more physiological pH 6.2 and with a broad range of methods [35]. Therefore, we studied the roles of the first six residues of β _2_M in the modulation of both β _2_M fibrillization and the seed-dependent propagation of β _2_M fibrils at acidic pH 2.5 and more physiological pH 6.2. Notably, we showed that at more physiological pH 6.2, amyloid fibrils were assembled *in vitro* only from recombinant, ΔN4, ΔN5, and ΔN6 β _2_M but not from WT β _2_M and its three truncated variants ΔN1 to ΔN3. One striking observation is that WT β _2_M fibril seeds only have a very weak ability to trigger fibril formation of ΔN6 (also ΔN5 and ΔN4) at acidic pH 2.5, possibly because hydrophobic interactions between WT β _2_M fibril core [27] and ΔN6 β _2_M (also ΔN5 and ΔN4 β _2_M) monomers are very weak. These cumulative results pointed to a critical role of the residues 4 to 6 (TPK) at the N-terminus in modulating amyloid fibril formation of β _2_M at acidic pH 2.5 and more physiological pH 6.2. Intriguingly, the interaction of β _2_M fibril seeds with β _2_M monomers has been found to regulate misfolding and aggregation of β _2_M [ 11, 13, 24, 29]. It has been reported that ΔN6 forms fibrils easily at neutral pH *in vitro* without any cosolvents or additives [ 25, 29, 30, 35]. Moreover, the resulted ΔN6 seeds are able to enhance the rate of fibril formation of WT β _2_M [ 11, 13, 29, 35].

We further discuss the role of flexible regions of proteins (in this case, the N-terminus of β _2_M) in amyloid fibril formation. ΔN6 β _2_M exhibited lower conformational stability than the wild-type protein, as observed by Esposito *et al*. [30] who also concluded (mostly by NMR) that the greater conformational flexibility resulting from the loss of the first six residues promotes the adoption of intermediate conformational states that facilitate aggregation and fibril formation. From the crystal structure (and NMR studies), the first six residues of β _2_M are flexible and unstructured [51], i.e. they are not part of the immunoglobulin β-sheet fold. Goldschmidt *et al*. [52] observed that unstructured regions in proteins often protect fibril-prone regions in proteins (that are buried within the tertiary structure of the protein) from aggregating. The first six residues in β _2_M fit these criteria. Their absence in ΔN6 β _2_M would facilitate access to the fibril-prone regions and hence enhance the protein’s aggregation propensity. The structure of β _2_M fibrils is now available [27]. Phe22 to Val85 form the fibril core which corresponds well to β-strands B-F in the crystal structure of monomeric, native β _2_M. Lys6 and Ser28 stabilize the β-sheet, and the remaining N- and C-terminal regions in the β _2_M fibrils are unstructured [27]. These data fit well with the involvement of destabilization/unfolding of the N-terminal region in fibril formation and are related to the results presented here.

Conformational stability of β _2_M fibrils can be assessed by measuring resistance to chemical and/or thermal denaturation [ 35, 53, 54], which may link to the progression of dialysis-related amyloidosis. Notably, we showed that the ΔN6 β _2_M fibril formed at pH 2.5 exhibited a significantly lower conformational stability than the WT β _2_M fibril formed under such conditions. Intriguingly, the ΔN6 fibril formed at pH 6.2 is also significantly less stable than the WT β _2_M fibril formed at pH 2.0 [35]. Thus, deletion of the N-terminal hexapeptide significantly decreases the conformational stability of β _2_M fibrils and amyloid fibrils formed by ΔN6 are conformationally distinct from that formed by WT β _2_M. Our data suggest that the instability property of ΔN6 fibrils may lead to an increased capability of propagation, which may be strictly related to the onset of dialysis-related amyloidosis.

In summary, we reported that the residues 4 to 6 (TPK) at the N-terminus in particular modulate amyloid fibril formation of β _2_M at more physiological pH 6.2. We proposed a hypothetical model, which shows how the six residues at the N-terminus modulate fibril propagation of β _2_M and the interactions of WT β _2_M with N-terminally truncated variants at pH 6.2 ( Figure 8H). The loss of the first six residues (ΔN6) facilitates amyloid fibril formation from ΔN6 β _2_M monomers, and ΔN6 fibril seeds are then formed after fragmentation. Under more physiological conditions (pH 6.2), we observed that homo- and heterotypic seeding of the ΔN6 fibrils strongly promoted amyloid fibril formation of WT β _2_M and its truncated variant ΔN6, indicating a strong seeding activity of ΔN6 fibrils. This study enhances our understanding of how the six residues at the N-terminus regulate amyloid formation and seed-dependent propagation of β _2_M and helps explain the mechanism underlying fibril propagation and spreading of β _2_M leading to dialysis-related amyloidosis.

## Supporting information

21246Supplemental_Data

## Supplementary Data

Supplementary data is available at *Acta Biochimica et Biophysica Sinica* online.
