# Supplementary material for: The residues 4 to 6 at the N-terminus in particular modulate fibril propagation of β 2-microglobulin : N-terminal modulates β2M fibril propagation
Source: Acta Biochim Biophys Sin (Shanghai). 2021 Dec 23;54(2):187–98. doi: 10.3724/abbs.2021017 (PMC9909321; doi:10.3724/abbs.2021017)
Supplement: 21246Supplemental_Data [file 21246Supplemental_Data.doc]

**Supplementary Table S1. The primers designed for N-terminal truncated variants of human β_2_**M

| **Name** | **Primer Sequence** |
| --- | --- |
| S-ΔN1 β_2_M | 5’-GAATTCCATATGCAGCGTACTCC-3’ |
| A-ΔN1 β_2_M | 5’-CCGCTCGAGTTACATGTCTCGATCC-3’ |
| S-ΔN2 β_2_M | 5’-GAATTCCATATGCGTACTCCAAAGATTC-3’ |
| A-ΔN2 β_2_M | 5’-CCGCTCGAGTTACATGTCTCGATCC-3’ |
| S-ΔN3 β_2_M | 5’-GAATTCCATATGACTCCAAAGATTCAGG-3’ |
| A-ΔN3 β_2_M | 5’-CCGCTCGAGTTACATGTCTCGATCC-3’ |
| S-ΔN4 β_2_M | 5’-GAATTCCATATGCCAAAGATTCAGG-3’ |
| A-ΔN4 β_2_M | 5’-CCGCTCGAGTTACATGTCTCGATCC-3’ |
| S-ΔN5 β_2_M | 5’-GAATTCCATATGAAGATTCAGGTTTACTC-3’ |
| A-ΔN5 β_2_M | 5’-CCGCTCGAGTTACATGTCTCGATCC-3’ |
| S-ΔN6 β_2_M | 5’-GAATTCCATATGATTCAGGTTTACTCACG-3’ |
| A-ΔN6 β_2_M | 5’-CCGCTCGAGTTACATGTCTCGATCC-3’ |


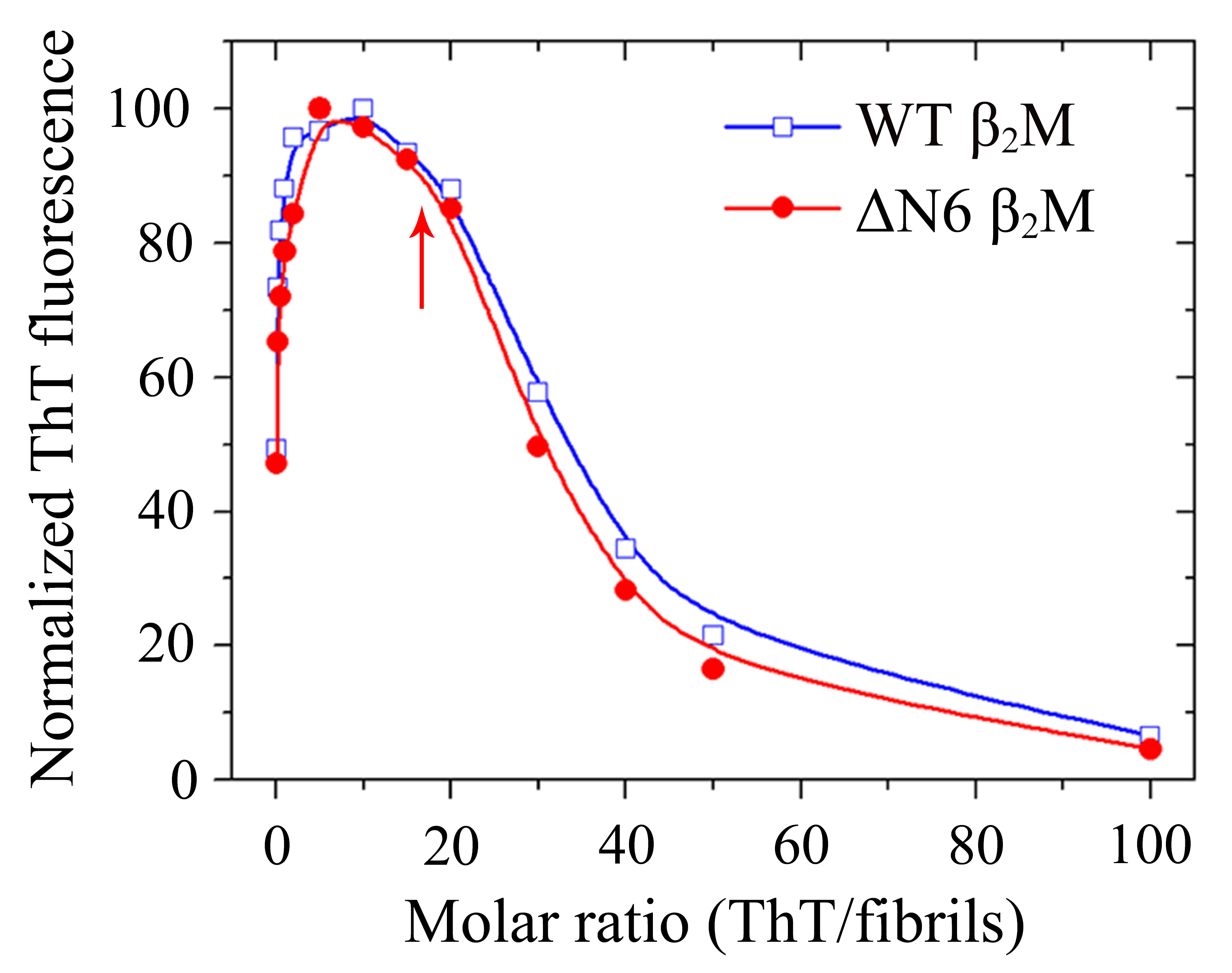


**Supplementary Figure S1.** **ThT (25 μM) did not quench the fluorescence of 1.42 μM β_2_M fibrils** Amyloid fibrils were produced from WT β_2_M and ΔN6 β_2_M incubated at pH 2.5 with agitation at 220 rpm and 37°C for 78 and 108 h, respectively. ThT concentration-dependent binding profiles were monitored by ThT fluorescence for amyloid fibrils produced from WT β_2_M (blue) and ΔN6 β_2_M (red) at pH 2.5, which were incubated for 5 min at 25°C with increasing concentrations of ThT. The final concentration of β_2_M fibrils was 1.42 μM. The condition of 25 μM ThT binding to 1.42 μM β_2_M is highlighted using a red arrow.


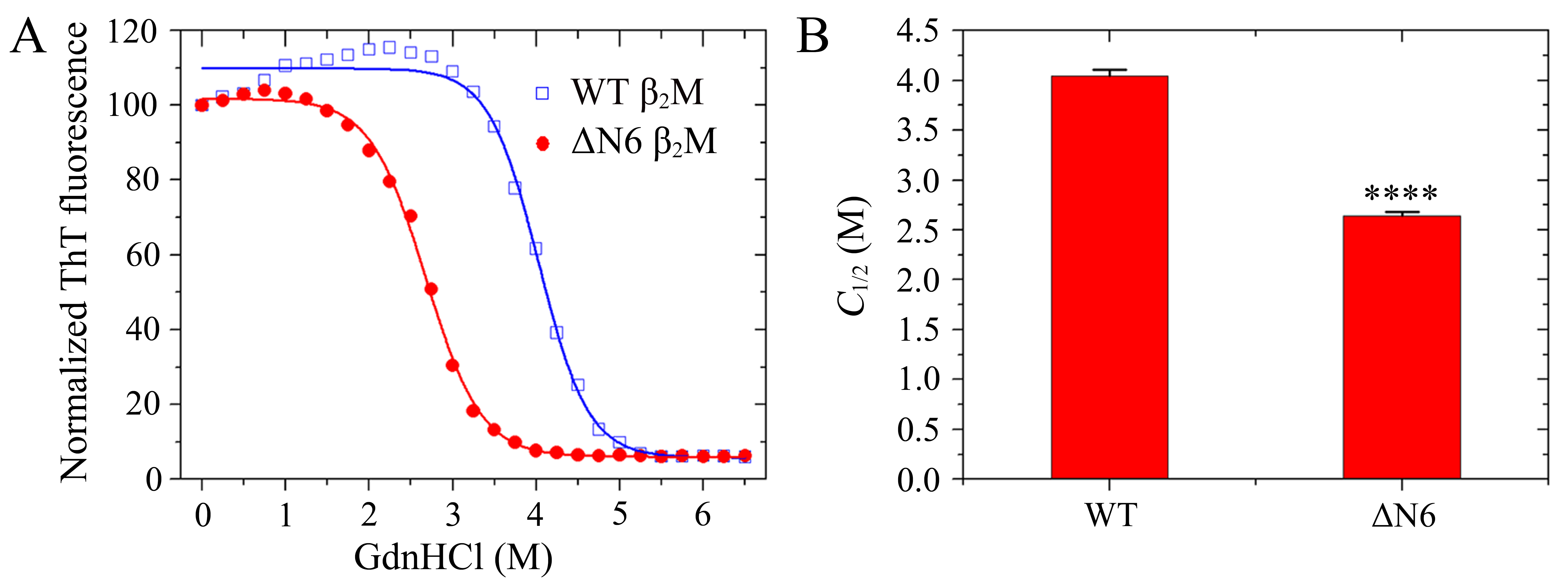


**Supplementary Figure S2. The ΔN6 truncation significantly decreased the conformational stability of β_2_M fibrils** Amyloid fibrils were produced from WT β_2_M and ΔN6 β_2_M incubated at pH 2.5 with agitation at 220 rpm and 37 °C for 78 and 108 h, respectively. (A) GdnHCl concentration-dependent denaturation profiles were monitored by ThT fluorescence for amyloid fibrils produced from WT β_2_M (blue) and ΔN6 β_2_M (red) at pH 2.5, which were incubated for 1 h at 25°C with increasing concentrations of GdnHCl. (B) The *C*_1/2_ values for amyloid fibrils of WT β_2_M and ΔN6 β_2_M were determined using a sigmoidal equation and are expressed as the mean ± SD of the values obtained from 3 independent experiments. *C*_1/2_, *P* = 0.0000049. The Student’s *t* test was used to perform statistical analyses. Values of *P* < 0.05 indicate statistically significant differences. **P*<0.05; ***P* < 0.01; ****P*< 0.001; and *****P* < 0.0001 relative to WT β_2_M (a control).


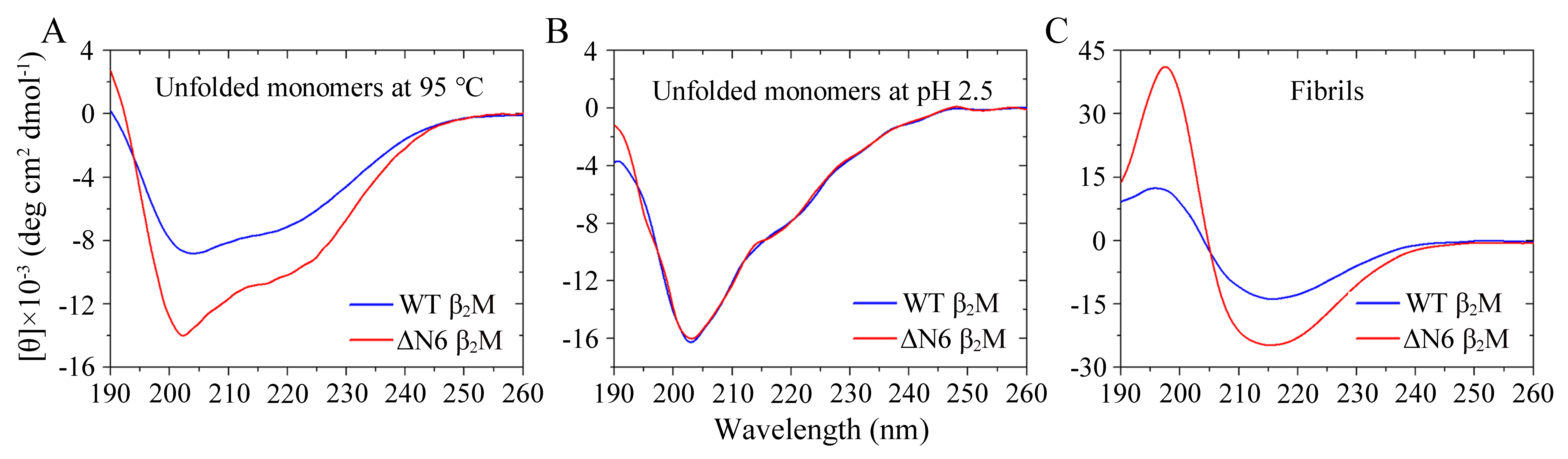


**Supplementary Figure S3. The unfolded monomers disassembled from** **β_2_M fibrils were distinguished by far-UV CD spectroscopy**  The secondary structures of the unfolded monomers disassembled from 25 μM fibrils of WT β_2_M (blue) and ΔN6 β_2_M (red) (A), the unfolded monomers of 25 μM WT β_2_M (blue) and ΔN6 β_2_M (red) induced by acid at pH 2.5 (B), and 25 μM fibrils of WT β_2_M (blue) and ΔN6 β_2_M (red) formed at pH 2.5 (C), monitored by far-UV CD. (A) The unfolded monomers at 95°C and (B) those induced by acid at pH 2.5 were both characterized by a largely random coil structure. (C) In contrast, the β_2_M fibrils at 25°C were characterized by β-sheet-rich structures.


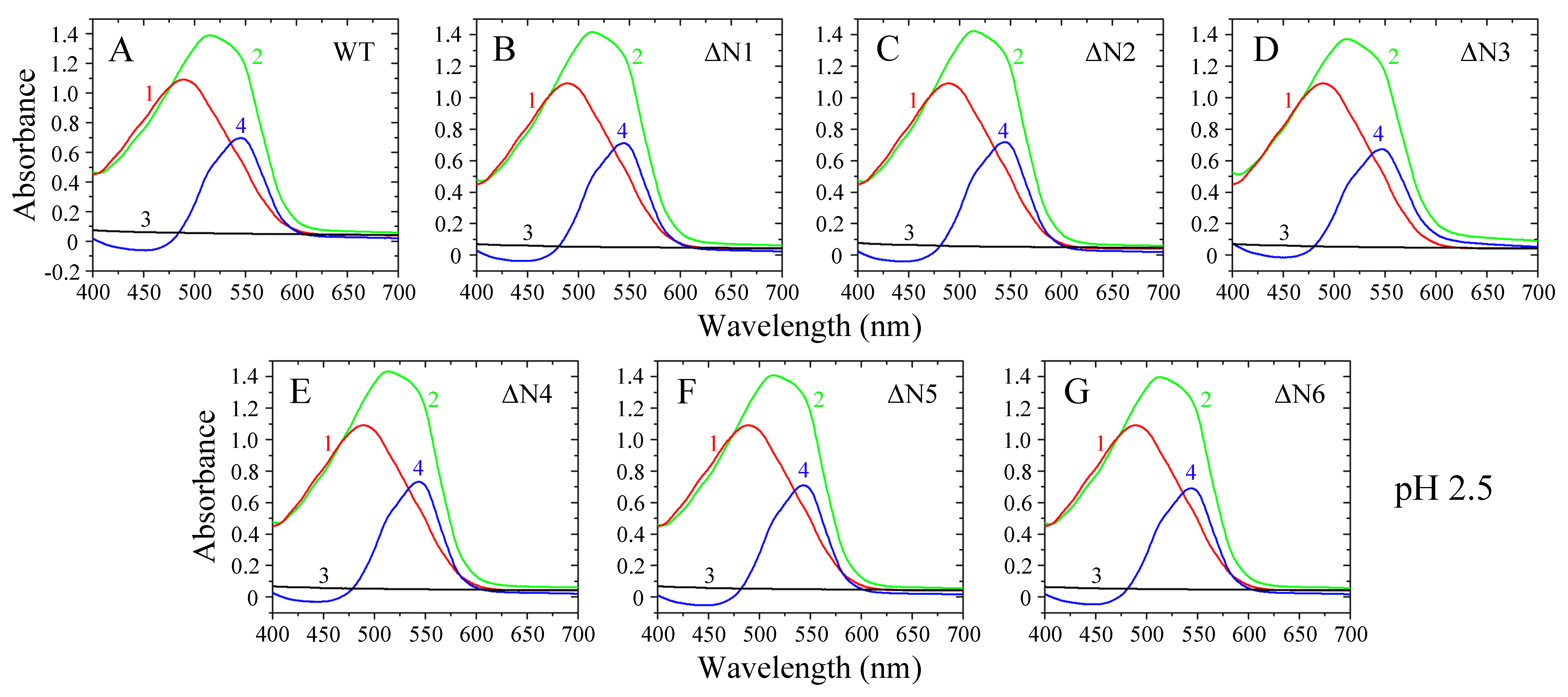


**Supplementary Figure S4. Amyloid fibrils were formed *in vitro* from recombinant, WT β_2_M and its six truncated variants at acidic pH 2.5, as revealed by Congo red binding assays** (A−G) Amyloid fibrils of wild-type (WT) β_2_M and its N-terminally-truncated variants were formed at pH 2.5 with agitation at 220 rpm and 37°C. Absorbance data are shown for amyloid fibrils at the end of fibril formation for 10 μM WT β_2_M (A) and its truncated mutants ΔN1 (B), ΔN2 (C), ΔN3 (D), ΔN4 (E), ΔN5 (F), and ΔN6 (G) in the presence of 50 μM Congo red at 25°C. The difference spectra (Curve 4, blue) with the maximum absorbance at 550 nm were obtained by subtracting the absorbance spectra of β_2_M fibrils alone (Curve 3, black) and Congo red alone (Curve 1, red) with the maximum absorbance at 490 nm from those of β_2_M fibrils + Congo red (Curve 2, green). At pH 2.5, we produced amyloid fibrils from WT β_2_M and its six truncated variants. All Congo red binding assays were repeated at least three times, and the results were reproducible.


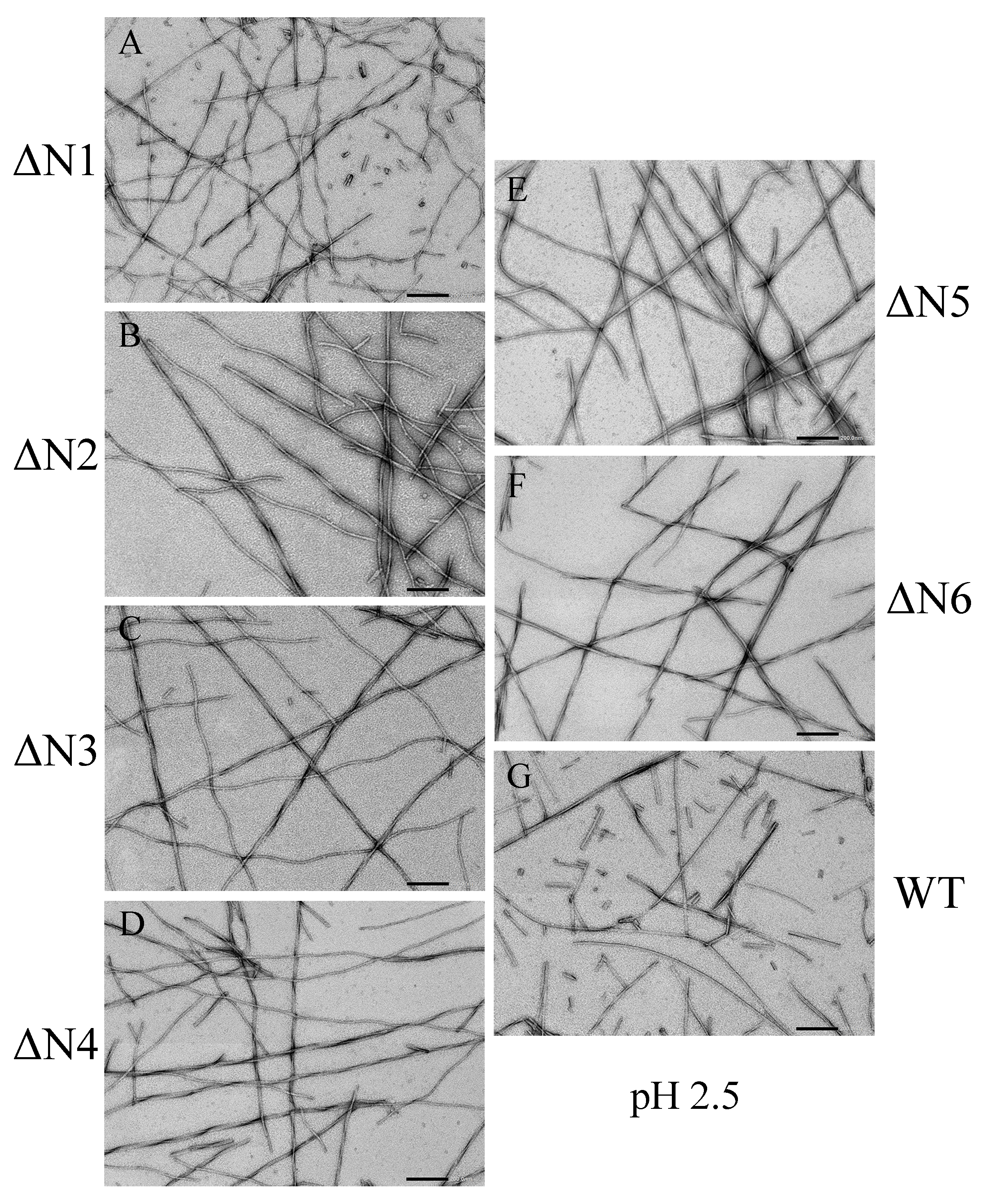


**Supplementary Figure S5.** **High-resolution TEM images of β_2_M fibrils at acidic pH 2.5** (A−G) Negative-staining TEM images of amyloid fibrils formed by WT β_2_M (G) incubated for 78 h, ΔN1 (A) and ΔN2 (B) incubated for 96 h, and ΔN3 (C), ΔN4 (D), ΔN5 (E), and ΔN6 (F) incubated for 108 h at pH 2.5 with agitation at 220 rpm and 37°C. A 2% (w/v) uranyl acetate solution was used for staining the fibrils negatively. Scale bar: 200 nm.


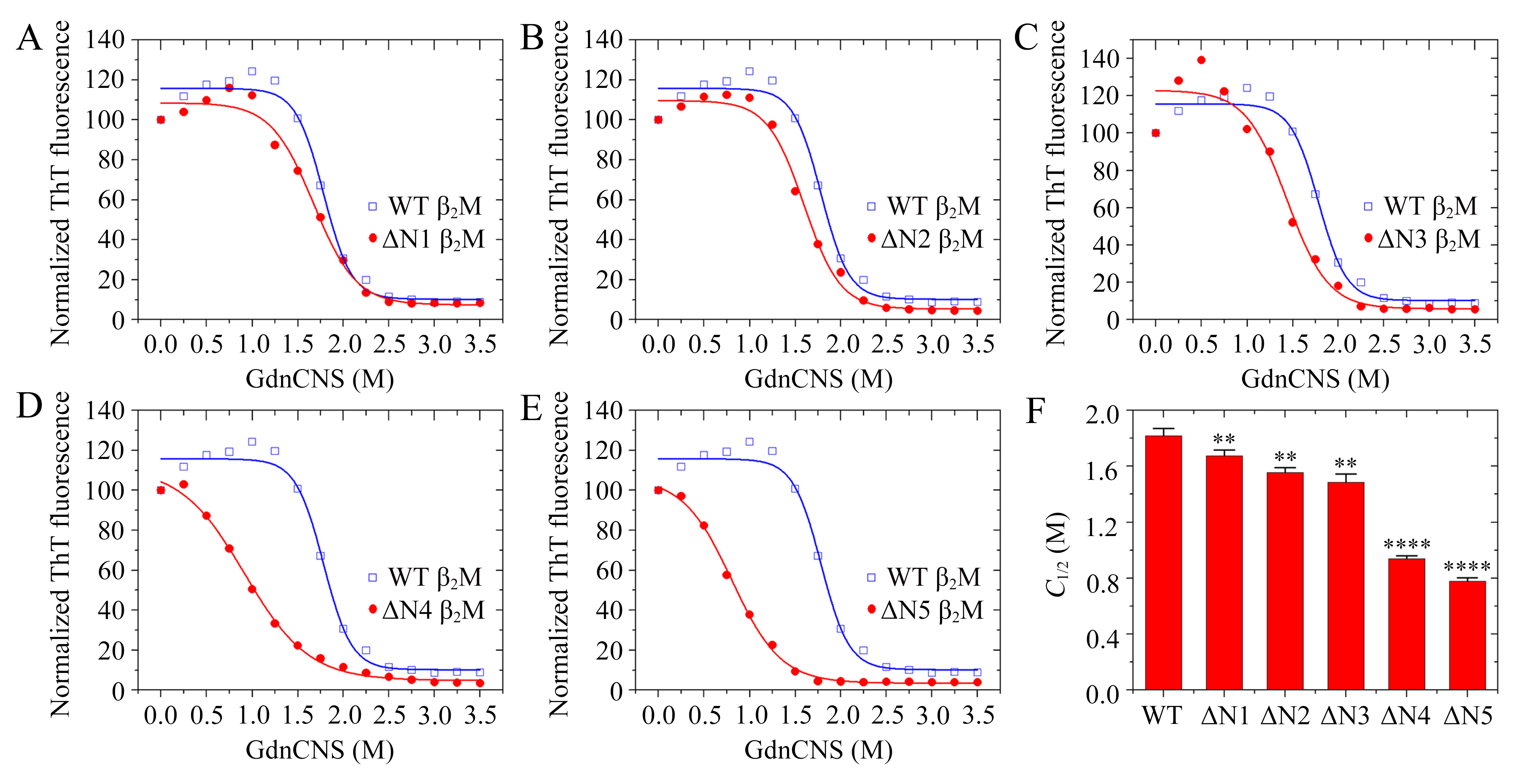


**Supplementary Figure S6.** **The ΔN5 to ΔN4 truncation decreased the conformational stability of β_2_M fibrils more significantly than the ΔN3 to ΔN1 truncation** Amyloid fibrils were produced from WT β_2_M, the truncated variants ΔN1 and ΔN2, and ΔN3 to ΔN5 incubated at pH 2.5 with agitation at 220 rpm and 37°C for 78, 96, and 108 h, respectively. GdnCNS concentration-dependent denaturation profiles monitored by ThT fluorescence for amyloid fibrils produced from WT β_2_M (blue) and ΔN1 β_2_M (red) (A), ΔN2 β_2_M (red) (B), ΔN3 β_2_M (red) (C), ΔN4 β_2_M (red) (D), and ΔN5 β_2_M (red) (E) at pH 2.5, which were incubated for 1 h at 25°C with increasing concentrations of GdnCNS. (F) The *C*_1/2_ values for amyloid fibrils of WT β_2_M and its five truncated variants were determined using a sigmoidal equation and are expressed as the mean ± SD of the values obtained from 3 independent experiments. *C*_1/2_, *P* = 0.0084, 0.0018, 0.0018, 0.000011, and 0.0000059 for ΔN1, ΔN2, ΔN3, ΔN4, and ΔN5, respectively. **P*<0.05; ***P* < 0.01; ****P*< 0.001; and *****P* < 0.0001 relative to WT β_2_M (a control).


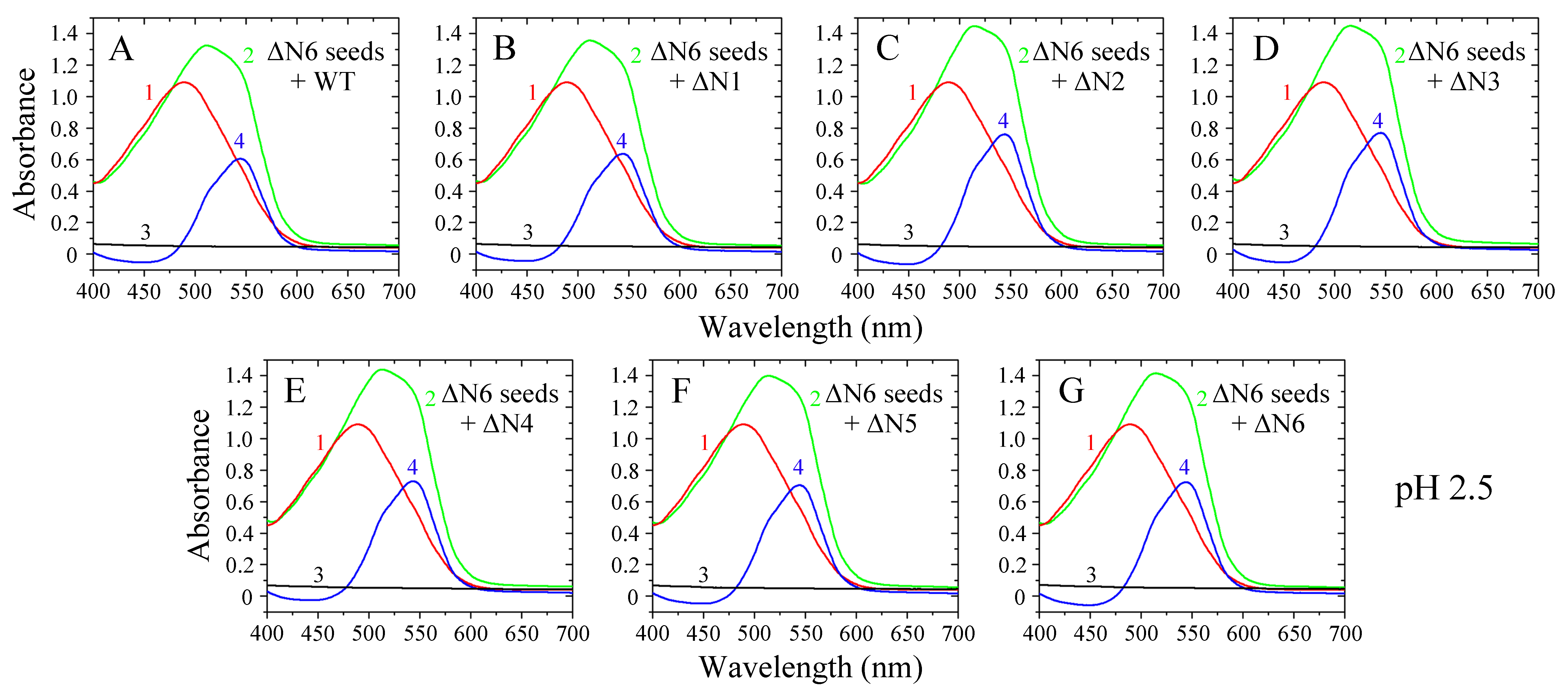


**Supplementary Figure S7.** **Homo- and heterotypic seeding of ΔN6 fibrils strongly promoted amyloid fibril formation of WT β_2_M and its six truncated variants at acidic pH 2.5, as revealed by Congo red binding assays** (A−G) Amyloid fibrils of WT β_2_M and its N-terminally-truncated variants were induced by 2% (v/v) ΔN6 fibril seeds at pH 2.5 with agitation at 220 rpm and 37°C. Absorbance data are shown for amyloid fibrils at the end of fibril formation for 10 μM WT β_2_M (A) and its truncated mutants ΔN1 (B), ΔN2 (C), ΔN3 (D), ΔN4 (E), ΔN5 (F), and ΔN6 (G) in the presence of 50 μM Congo red at 25°C. The difference spectra (Curve 4, blue) with the maximum absorbance at 550 nm were obtained by subtracting the absorbance spectra of β_2_M fibrils alone (Curve 3, black) and Congo red alone (Curve 1, red) with the maximum absorbance at 490 nm from those of β_2_M fibrils + Congo red (Curve 2, green). All Congo red binding assays were repeated at least three times, and the results were reproducible.


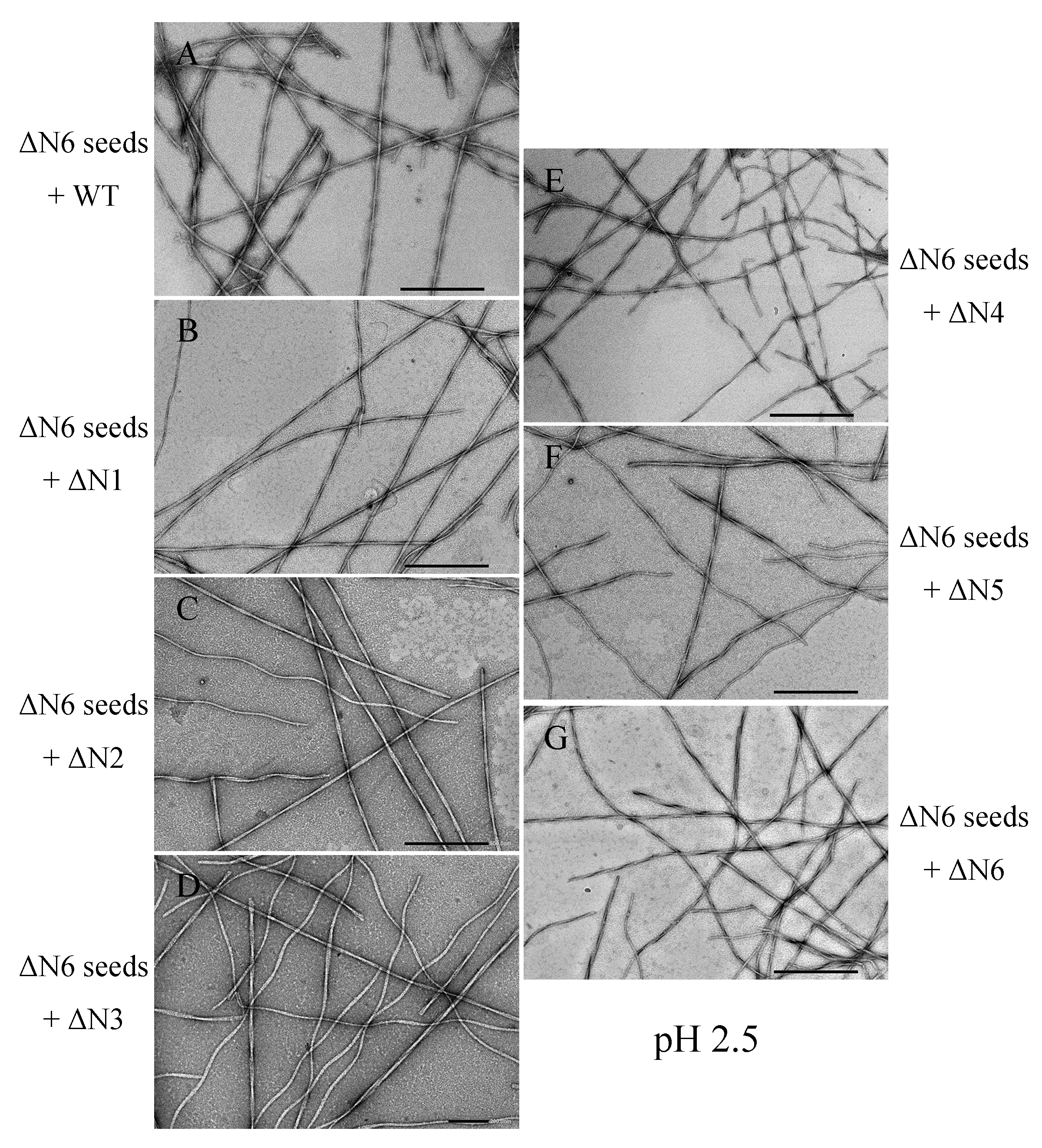


**Supplementary Figure S8.** **TEM images** **of β_2_M fibrils induced by ΔN6 fibril seeds at acidic pH 2.5** (A−G) Negative-staining TEM images of seed-dependent propagation for WT β_2_M (A), ΔN1 (B), ΔN2 (C), ΔN3 (D), ΔN4 (E), ΔN5 (F), and ΔN6 (G) incubated with 2% (v/v) ΔN6 fibril seeds at pH 2.5 with agitation at 220 rpm and 37°C for 10 h. Scale bar: 500 nm. Homo- and heterotypic seeding of ΔN6 fibrils strongly promoted amyloid fibril formation of WT β_2_M and its six truncated variants at pH 2.5.


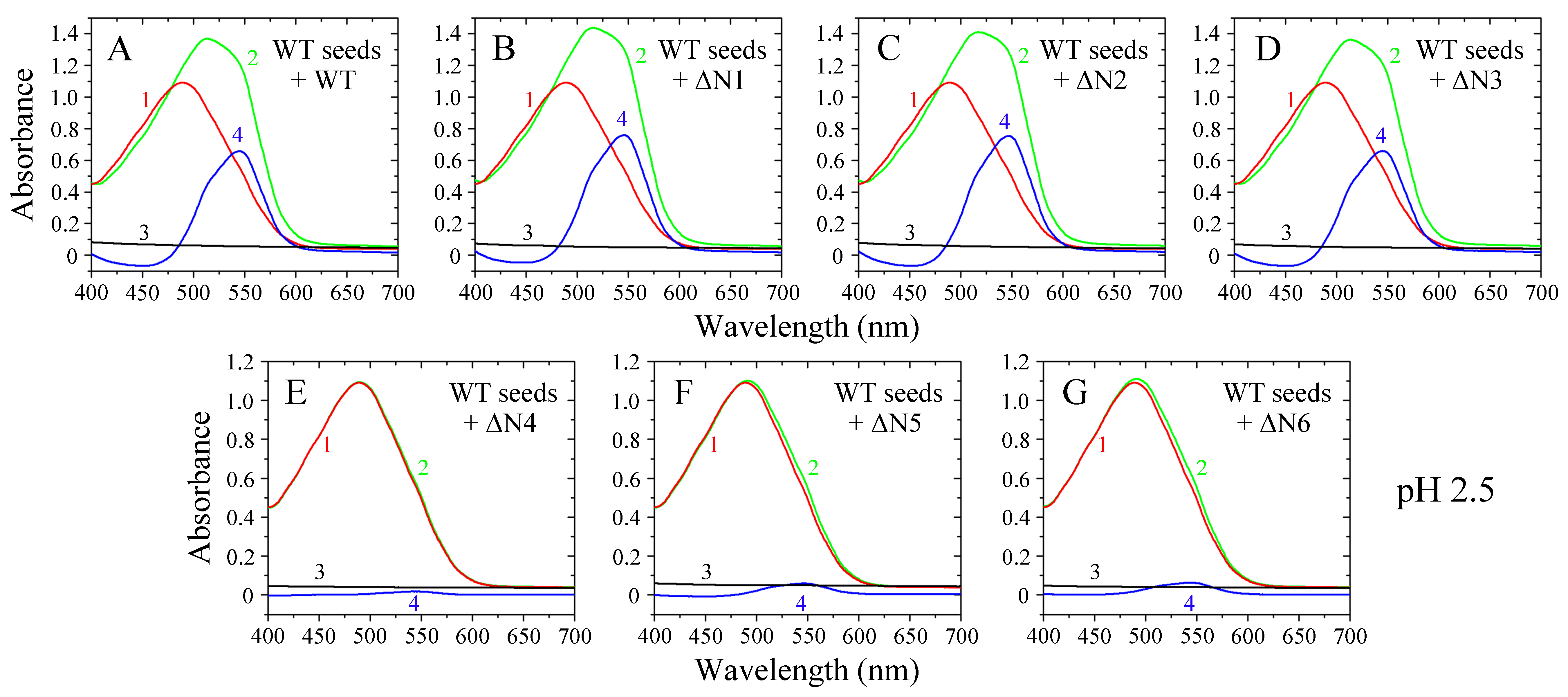


**Supplementary Figure S9.** **Homo- and heterotypic seeding of WT β_2_M fibrils facilitated amyloid fibril formation of the wild-type protein and its three truncated variants ΔN1 to ΔN3 but not ΔN4, ΔN5, and ΔN6 at acidic pH 2.5, as revealed by Congo red binding assays**  (A−G) Amyloid fibrils of WT β_2_M and its N-terminally-truncated variants were induced by 2% (v/v) WT β_2_M fibril seeds at pH 2.5 with agitation at 220 rpm and 37°C. Absorbance data are shown for amyloid fibrils at the end of fibril formation for 10 μM WT β_2_M (A) and its truncated mutants ΔN1 (B), ΔN2 (C), ΔN3 (D), ΔN4 (E), ΔN5 (F), and ΔN6 (G) in the presence of 50 μM Congo red at 25°C. The difference spectra (Curve 4, blue) with the maximum absorbance at 550 nm were obtained by subtracting the absorbance spectra of β_2_M fibrils alone (Curve 3, black) and Congo red alone (Curve 1, red) with the maximum absorbance at 490 nm from those of β_2_M fibrils + Congo red (Curve 2, green). All Congo red binding assays were repeated at least three times, and the results were reproducible.
